# Head and neck cancer treatment outcome prediction: a comparison between machine learning with conventional radiomics features and deep learning radiomics

**DOI:** 10.3389/fmed.2023.1217037

**Published:** 2023-08-30

**Authors:** Bao Ngoc Huynh, Aurora Rosvoll Groendahl, Oliver Tomic, Kristian Hovde Liland, Ingerid Skjei Knudtsen, Frank Hoebers, Wouter van Elmpt, Eirik Malinen, Einar Dale, Cecilia Marie Futsaether

**Affiliations:** ^1^Faculty of Science and Technology, Norwegian University of Life Sciences, Ås, Norway; ^2^Department of Circulation and Medical Imaging, Norwegian University of Science and Technology, Trondheim, Norway; ^3^Department of Medical Physics, Oslo University Hospital, Oslo, Norway; ^4^Department of Radiation Oncology (MAASTRO), Maastricht University Medical Center, Maastricht, Netherlands; ^5^GROW School for Oncology and Reproduction, Maastricht University Medical Center, Maastricht, Netherlands; ^6^Department of Physics, University of Oslo, Oslo, Norway; ^7^Department of Oncology, Oslo University Hospital, Oslo, Norway

**Keywords:** machine learning, deep learning, artificial intelligence, feature selection, radiomics, head and neck cancer, interpretability, outcome prediction

## Abstract

**Background:**

Radiomics can provide in-depth characterization of cancers for treatment outcome prediction. Conventional radiomics rely on extraction of image features within a pre-defined image region of interest (ROI) which are typically fed to a classification algorithm for prediction of a clinical endpoint. Deep learning radiomics allows for a simpler workflow where images can be used directly as input to a convolutional neural network (CNN) with or without a pre-defined ROI.

**Purpose:**

The purpose of this study was to evaluate (i) conventional radiomics and (ii) deep learning radiomics for predicting overall survival (OS) and disease-free survival (DFS) for patients with head and neck squamous cell carcinoma (HNSCC) using pre-treatment ^18^F-fluorodeoxuglucose positron emission tomography (FDG PET) and computed tomography (CT) images.

**Materials and methods:**

FDG PET/CT images and clinical data of patients with HNSCC treated with radio(chemo)therapy at Oslo University Hospital (OUS; *n* = 139) and Maastricht University Medical Center (MAASTRO; *n* = 99) were collected retrospectively. OUS data was used for model training and initial evaluation. MAASTRO data was used for external testing to assess cross-institutional generalizability. Models trained on clinical and/or conventional radiomics features, with or without feature selection, were compared to CNNs trained on PET/CT images without or with the gross tumor volume (GTV) included. Model performance was measured using accuracy, area under the receiver operating characteristic curve (AUC), Matthew’s correlation coefficient (MCC), and the F1 score calculated for both classes separately.

**Results:**

CNNs trained directly on images achieved the highest performance on external data for both endpoints. Adding both clinical and radiomics features to these image-based models increased performance further. Conventional radiomics including clinical data could achieve competitive performance. However, feature selection on clinical and radiomics data lead to overfitting and poor cross-institutional generalizability. CNNs without tumor and node contours achieved close to on-par performance with CNNs including contours.

**Conclusion:**

High performance and cross-institutional generalizability can be achieved by combining clinical data, radiomics features and medical images together with deep learning models. However, deep learning models trained on images without contours can achieve competitive performance and could see potential use as an initial screening tool for high-risk patients.

## 1. Introduction

Head and neck cancer (HNC) accounts for 3% of cancers worldwide (1). The majority of HNCs are head and neck squamous cell carcinomas (HNSCC) of the oral cavity, oropharynx, hypopharynx and larynx (2, 3). Most patients present with locally advanced disease where standard treatment is concurrent radio-chemotherapy with or without surgery first (4). Pre-treatment imaging is routinely done using computed tomography (CT) and/or magnetic resonance imaging (MRI), but ^18^F-fluorodeoxyglucose positron emission tomography (FDG PET)/CT can be superior at identifying locoregional nodal involvement or distant metastasis and cancer recurrence at follow-up (5).

Major risk factors for HNSCC include smoking and heavy alcohol consumption particularly for oral cavity, hypopharyngeal, and laryngeal cancers, as well as oropharyngeal cancers not related to human papillomavirus (HPV) (5, 6). HPV is associated with an estimated 60%–70% of oropharyngeal cancers. The 8th edition of the American Joint Committee on Cancer (AJCC) tumor–node–metastasis staging system (TNM8) defines HPV-related and HPV-unrelated oropharyngeal cancer as distinct entities with different tumor characteristics and treatment outcomes (6). Staging is strongly associated with treatment outcome as are other clinical factors such as comorbidity status, tobacco use, gender, and age (5). Image-based parameters such as the FDG PET maximum standardized uptake value (SUV_max_) (7) of the primary tumor (8) or texture-related parameters (9) characterizing tumor heterogeneity (10) may provide additional information aiding in HNC outcome prediction.

Image-based parameters can be obtained using radiomics where medical images are mined for information not readily apparent to the human eye that can improve and guide diagnostics and medical decision-making (9, 11–14). In its basic form, radiomics uses mathematical algorithms to convert 2D or 3D images into high-dimensional tabular data of radiomics features. These features require contouring of the region of interest (ROI), such as the primary tumor and involved lymph nodes, and consist of three main categories describing the intensity distribution, the shape and size, and the texture of the ROI (9). The tabular data is passed to a machine learning algorithm for model training and prediction of a clinical endpoint and is henceforth referred to as conventional radiomics. In deep learning radiomics, on the other hand, images are sent directly into a convolutional neural network (CNN) either with tumor/node contours or without these contours (9, 15). Thus, calculation of radiomics features can be bypassed, as can tumor and node contouring. During training, the network automatically learns discriminant features which form the basis for its prediction.

For HNSCC, radiomics has been used for prediction of treatment outcomes including locoregional control (LRC), distant metastases (DM), disease-free survival (DFS), progression-free survival (PFS), and overall survival (OS) (15–22), as well as nodal failure (23, 24), HPV status (10), and xerostomia (25). Radiomics features from multi-modality images can improve radiomics model performance relative to single-modality features in some but not all cases (19, 26). Other studies find that radiomics features alone do not improve model performance relative to clinical models, but combined models incorporating radiomics, clinical and biological features can significantly improve performance (19, 20, 23, 24). Single and multi-modality deep learning radiomics have also been explored for HNC and provide overall good performance, on par with or higher than conventional radiomics (15, 27).

Despite its potential, radiomics has certain pitfalls. Radiomics is influenced by several factors in the radiomics pipeline that can affect both the robustness and generalizability of models across patient cohorts and centers (12, 28). These include differences between image acquisition and reconstruction protocols, uncertainties introduced due to interobserver variations during contouring of the ROI, as well as the many possibilities available for image pre-processing (29). In addition, conventional radiomics involves several alternatives related to image discretization and feature extraction (29), which can generate thousands of features that may greatly outnumber the number of patients. An exhaustive search for all possible combinations of relevant features is not possible and may end up with an overfitted solution where the set of features is too focused on the training or validation data, while not generalizing well to unseen test data, preferably from an external center (11). An optimal feature selection method should find a small set of features that are representative across the entire patient population, are medically justifiable, and have diagnostically discriminant properties. This would greatly improve model interpretability. In addition, although powerful, deep learning radiomics can be much more difficult to interpret relative to models based on a few interpretable radiomics features (30–33).

As conventional radiomics may be particularly sensitive to issues in the radiomics pipeline, we hypothesize that deep learning radiomics may be more robust and generalizable to external testing and provide higher overall performance. We further hypothesize that combining explainable artificial intelligence (AI) with deep learning radiomics will provide a sanity check that deep learning models focus on regions within the images that are of importance for treatment outcome. The aim of this study was therefore to conduct a comprehensive comparison of prediction models using conventional radiomics or deep learning radiomics based on pre-treatment multi-modal PET/CT of HNC for two clinical endpoints OS and DFS. Models based solely on clinical factors were used as reference to assess the added benefit of radiomics. To assess model generalizability, prediction models were first trained and tested on internal data from one center and then tested on external data from another center in a different country.

## 2. Materials and methods

### 2.1. Patient characteristics

In this study, two HNSCC datasets from two separate centers, namely Oslo University Hospital (OUS) and Maastricht University Medical Center (MAASTRO), were analyzed. The OUS patients were used for model training, validation and internal testing, whereas the MAASTRO patients were used as an external test set. HNSCC patients treated with radio(chemo)therapy at OUS between 2007 and 2013, or MAASTRO between 2008 and 2014, were retrospectively collected. The OUS patient cohort is described in detail in Moan et al. (7) which assessed the prognostic role of clinical factors and standard FDG PET parameters on DFS. Patients from the OUS cohort have also been analyzed in three automatic segmentation studies (34–36). Briefly, the inclusion criteria at both centers were: HNSCC of the oral cavity, oropharynx, hypopharynx and larynx, and available radiotherapy plans based on FDG PET/CT. Patients who did not have a contrast-enhanced CT along with the PET examination, as well as patients with oropharyngeal cancer and unknown HPV status, were excluded from the present study. This resulted in 139 OUS patients and 99 MAASTRO patients included for analysis. Characteristics of the included patients are summarized in Table 1. Due to the moderate number of included patients and the different primary tumor site distributions between the OUS and MAASTRO datasets (Table 1), a mixed analysis, i.e., including all primary tumor sites in the datasets, was preferred over subgroup analysis, i.e., focusing on one single primary tumor site. Mixed analysis for HNC outcome prediction is also encountered frequently in the literature, as summarized in Adeoye et al. (37).

**Table 1:** Patient characteristics of the OUS and MAASTRO datasets.

Characteristics	OUS (*n* = 139)	MAASTRO (*n* = 99)	*p*-value^c^
*Age (years)*			
Mean ± SD (median)	60.2 ± 7.7 (60)	61.6 ± 9.5 (61)	ns
*Gender*			
Female	32 (23.0%)	26 (26.3%)	ns
Male	107 (77.0%)	73 (73.7%)	
*Smoking (pack years)*			
Mean ± SD (median)	25.0 ± 22.8 (22.5)	46.1 ± 47.5 (40.0)	<0.0001
*Tumor site*			
Oral cavity	11 (7.9%)	3 (3.0%)	ns
Oropharynx	91 (65.5%)	44 (44.4%)	<0.01
Hypopharynx	16 (11.5%)	15 (15.2%)	ns
Larynx	21 (15.1%)	37 (37.4%)	<0.001
*Overall stage (TNM8* *^a^* *)*			
I–II	72 (51.8%)	19 (19.2%)	<0.0001
III–IV	67 (48.2%)	80 (80.8%)	
*HPV-related* *^b^*			
Yes	80 (57.6%)	22 (22.2%)	<0.0001
No	59 (42.4%)	77 (77.8%)	
*Histologic grade*			
Low/moderate	43 (30.9%)	59 (59.6%)	<0.0001
High	96 (69.1%)	40 (40.4%)	
*Charlson comorbidity index*			
0	86 (61.9%)	25 (25.3%)	<0.0001
1–6	53 (38.1%)	74 (74.7%)	
*SUV_peak_*			
Mean ± SD (median)	11.0 ± 5.4 (10.0)	11.2 ± 6.2 (10.6)	ns
*MTV*			
Mean ± SD (median)	11.9 ± 13.5 (7.1)	15.1 ± 12.0 (10.9)	<0.001
*TLG*			
Mean ± SD (median)	121.0 ± 194.7 (56.6)	109.9 ± 105.6 (74.1)	0.03
*DFS*			
Event (class 1)	68 (48.9%)	59 (59.6%)	ns
Non-event (class 0)	71 (51.1%)	40 (40.4%)	
*OS*			
Event (class 1)	57 (41.0%)	53 (53.5%)	ns
Non-event (class 0)	82 (59.0%)	46 (46.5%)	

OUS, Oslo University Hospital; MAASTRO, Maastro Clinic, Maastricht; HPV, human papillomavirus; MTV, metabolic tumor volume; TLG, total lesion glycolysis; DFS, disease-free survival; OS, overall survival; ns, not significant.

a According to the 8th edition tumor—node—metastasis (TNM) system.

b HPV-related defined as HPV positive oropharyngeal cancers.

c *p*-values for unpaired Wilcoxon rank sum tests (continuous variables) and two-proportion *z*-tests (categorical variables) assessing the similarity in patient characteristics between the two cohorts (significance level: 0.05).

Both the OUS and MAASTRO patients were originally staged according to the 7th edition AJCC TNM system. The patients were, however, re-staged in accordance with the latest 8th edition TNM system, i.e., TNM8 (38). The similarity in patient characteristics between the two cohorts was assessed using unpaired Wilcoxon rank sum tests for continuous variables, and two-proportion *z*-tests for categorical variables. The statistical analysis was conducted in R. All tests were two-sided with a significance level of 0.05. An overview of the treatments given to the included patients at each center is provided in Supplementary Table A1.

For both datasets, the tabular data (i.e., clinical factors and response variables), as well as the image and contour data were screened for outliers and missing records prior to analysis. No imputation was performed.

The study was conducted in accordance with the Declaration of Helsinki. Approval was obtained from the Institutional Review Board and the Regional Ethics Committee for Medical and Health Research Ethics.

### 2.2. FDG PET/CT imaging and manual contouring

FDG PET/CT imaging was conducted at baseline following the standard image acquisition and reconstruction protocols used for HNC radiotherapy planning at each center. Briefly, imaging was performed using a radiotherapy compatible flat table with head support and a radiotherapy fixation mask. The included PET data were collected from the skull base to the mid chest with arms down. CT imaging was optimized for the head and neck region and performed with an iodinated contrast medium. The PET and CT were acquired in one session on the PET/CT scanner. Further details on the imaging protocols can be found in Supplementary Table A2.

The gross primary tumor (GTVp) and any involved nodal volume (GTVn) were contoured manually at the time of initial radiotherapy planning, in accordance with the local delineation protocols. For patients treated at OUS, contouring was done in accordance with the previous DAHANCA guidelines (39) based on the radiotherapy FDG PET/CT information using the following strategy: first an experienced nuclear medicine physician contoured the structures based on PET. Next, one or two oncology residents refined the delineations based on clinical information and the contrast-enhanced CT. Finally, a senior oncologist reviewed and approved the contours. For patients treated at MAASTRO, the GTVp and GTVn were delineated on the FDG PET/CT used for radiotherapy planning purposes, by the treating radiation oncologist with consultation of the nuclear medicine physician if needed. The contours were always reviewed and approved by a second radiation oncologist.

The PET/CT image series and DICOM structures were exported to an external computer and pre-processed using Interactive Data Language (IDL) v8.5 (Harris Geospatial Solutions, Broomfield, CO, United States). The PET and CT images along with the delineated GTVp and GTVn structures were resampled to 1 mm^3^ isotropic voxels and registered to a common frame of reference. PET image values (Bq/mL) were converted to standardized uptake values (SUV), normalized with respect to body weight.

### 2.3. Clinical factors and image features

The 11 clinical factors (7 factors + 4 tumor sites) listed in Table 1 were included for analysis. HPV-related HNSCC was defined as HPV positive oropharyngeal cancers. In addition, the three standard PET parameters SUV_peak_, metabolic tumor volume (MTV) and total lesion glycolysis (TLG) were calculated within the delineated GTVp. SUV_peak_ was defined as the maximum mean SUV of a 1 cm^3^ sphere with center within the GTVp. The MTV was thresholded within the GTVp using a threshold value equal to 50% of the SUV_peak_, whereas the TLG was defined as the MTV × SUV_mean_ where SUV_mean_ was defined as the mean SUV within the GTVp.

The PET/CT images and binary GTVp and GTVn image masks were also included for analysis. In addition, 354 radiomics features (40 first order, 14 shape, and 300 texture features) were extracted from the primary tumor based on the PET/CT images and the GTVp image masks using our in-house software *imskaper*^1^ based on PyRadiomics (40). As an extension to radiomics features from PyRadiomics, *imskaper* also extracted 20 local binary pattern (LBP) features (41) that capture additional 3D textures and patterns. Note that, all these 374 radiomics features were extracted from the primary tumor (GTVp) only, and not the involved nodal volume (GTVn). Detailed information about the process of extracting radiomics features can be found in Supplementary material B.

In summary, three different types of input data (D) were analyzed: tabular data including the aforementioned 11 clinical factors and three standard PET parameters (D1, 14 features); image-based tabular data of radiomics features (D2, 374 features); and image data including PET/CT images, the GTVp image masks, and the GTVn image masks (D3).

### 2.4. Data pre-processing

Before feeding to a conventional machine learning or deep learning algorithm, the tabular data (D1 and D2) was pre-processed. First, the clinical factors gender, histologic grade, Charlson comorbidity index, and TNM8 stage (Table 1) were converted into binary features. The dichotomization of the multi-level clinical factors histologic grade, Charlson comorbidity index and TNM8 is outlined in Table 1 and was done in accordance with a previous analysis of the same OUS cohort (7). Thereafter, since conventional machine learning and deep learning algorithms can only handle numeric data, the categorical feature tumor site was transformed into four different binary features representing each associated tumor site. Thus, D1 contained a total of 14 features, including 11 clinical factors and three standard PET parameters. Finally, all continuous features in D1 and D2 were standardized using the *z*-score standardization. In addition, we removed any duplicated radiomics features extracted from the PET and CT images (and from different binning settings), resulting in D2 having a total of 374 radiomics features. For details see Supplementary material B.

The original image size was reduced by cropping all images and structure masks to a 191 x 265 x 173 mm^3^ volume of interest, encompassing the head and neck region. A narrow soft-tissue CT window of center 70 Hounsfield Units (HU) and width 200 HU was applied to all CT images, in line with our previous analysis of the OUS patients (34, 35). Based on the 95% percentile of the maximum SUV (SUV_max_) among all patients in the OUS dataset (used for model training), we decided to apply a cut-off SUV of 25 on the PET images to remove any unexpected outliers. All voxel intensities of the CT and PET images were then scaled to the range [0, 1] before feeding into any CNN.

### 2.5. Modeling overview and response variables

As briefly outlined in the Introduction, we compared conventional radiomics to deep learning radiomics using the models shown in Table 2. For the conventional radiomics analysis we compared conventional machine learning prediction algorithms to fully connected neural networks (FCNN). For deep learning radiomics, we used CNNs and assessed the effect of including manually delineated GTVp and GTVn contours on model performance. Ensemble models combining both clinical and radiomics models were used to examine their relative importance for outcome prediction. Lastly, as a sanity check, we explored model explainability using a feature selection method based on a repeated elastic net technique (42) on radiomics features and explainable AI techniques for our CNN models.

**Table 2:** Overview of the models. The models (M1-M7, column 1) are sorted with increasing level of complexity. Input data (D) to each model is specified in the last two columns.

ID	Model architecture	Model type	Input data type	Input data
M1	Logistic regression	Conventional machine learning	Tabular data	Clinical data (D1), Radiomics data (D2) or all tabular data (D1 + D2)
M2	Random forest	Conventional machine learning	Tabular data	D1, D2 or D1 + D2
M3	Neural network without interaction	Deep learning (FCNN)	Tabular data	D1, D2 or D1 + D2
M4	Neural network with interaction	Deep learning (FCNN)	Tabular data	D1, D2 or D1 + D2
M5	EfficientNet3D CNN	Deep learning (CNN)	Image data	PET/CT (D3)
M6	EfficientNet3D CNN	Deep learning (CNN)	Image data	PET/CT & GTVp (D3)
M7	EfficientNet3D CNN	Deep learning (CNN)	Image data	PET/CT, GTVp & GTVn (D3)

FCNN, fully connected neural network; CNN, convolutional neural network.

Separate prediction models were trained to predict OS and DFS. OS was recorded as the length of time from start of treatment to death, and DFS was recorded as the length of time, from start of treatment, that the patients survived without any signs or symptoms of cancer. Both endpoints were treated as binary responses (0 and 1), in which class 1 indicated an event occurrence. Thus, for OS death was counted as an event, whereas for DFS local, regional or metastatic failure, or death, was counted as an event. End of follow-up time was June 13, 2017, for the OUS patients, and February 18, 2018, for the MAASTRO patients.

### 2.6. Feature selection using RENT—repeated elastic net technique

Given the high number of features in the tabular data there is the potential that machine learning models trained on these data will overfit, which in turn leads to poorer generalization capability when used on new unseen data. Moreover, high numbers of features may impact model interpretability, which is particularly problematic in healthcare data science, since interpretability is of highest importance to healthcare personnel. For this reason, we applied the repeated elastic net technique feature selection method named RENT (42) to the data using the RENT feature selection package (43). RENT acquires information on selection stability for each feature and utilizes this information for the selection of the final set of features. RENT, or slight modifications thereof, has been shown to be useful specifically for high-dimensional datasets consisting of more features than samples and has been applied in life-science research (44–46).

RENT trains an ensemble of generalized linear models on unique subsets of the data using elastic net regularization. The RENT framework provides information on which features in the dataset are selected consistently across all ensemble models and determines the final set of selected features based on the weight distribution of each feature. The distribution of the weights of each feature is acquired from the ensemble of models.

Three different weight-based criteria are used to summarize consistency for each feature across the modeled subsets: (i) the number of non-zero occurrences (how often was the feature weight non-zero, meaning that the feature been selected), (ii) the sum of coefficient signs (how often do the weights have the same sign?), and (iii) the Student’s t-test for deviation from zero (are the weights significantly larger than zero?). Each of the criteria emphasizes different aspects of stability and contribution. By thresholding each criterion and combining them, feature selection of higher quality than simple filter-based selection methods can be obtained.

In the feature selection process, we applied RENT in a brute-force manner by using repeated stratified *K*-fold cross validation with 5 splits and 20 repetitions with various combinations of values of the elastic net hyperparameter and regularization strength hyperparameter of the underlying logistic regression model used in RENT. For each combination of hyperparameters this results in a total of 100 sets of RENT-selected features where important features appear in all 100 feature sets (selection frequency 100%), non-informative features are present in none of the 100 feature sets (selection frequency 0%), and partially important features are present in between 0 and 100% of the 100 feature sets. The combination of hyperparameters leading to the best average predictive performance across 100 RENT models was then used for feature selection.

### 2.7. Prediction models

In this study, we conducted a comprehensive comparison of conventional machine learning and deep learning models based on different input types (D1, D2, and D3) to evaluate the effect of algorithm and input type on model performance and generalizability. Table 2 shows the seven models (M1–M7) with increasing complexity levels and different input data used in this study. The included classification algorithms (conventional machine learning: logistic regression and random forest; deep learning: fully CNN (FCNN) with or without interactions and EfficientNet CNN) were selected to span several levels of complexity. More specifically, the rationale for selecting logistic regression and random forest were as follows: (i) both algorithms are commonly used and have documented adequate performance for binary classification tasks, both outside and within the medical domain. (ii) Logistic regression is often a method of choice for outcome prediction within the medical domain, making it a natural reference algorithm. (iii) The two algorithms capture different relationships between the input features and the response variable: logistic regression is a linear algorithm resulting in linear decision boundaries between classes, whereas random forest can capture non-linear relationships, thereby resulting in more complex decision boundaries. The EfficientNet CNN was selected as it is one of the state-of-the-art 2D image classification models, with its ability to efficiently learn different characteristics from the images with significantly fewer parameters than similar CNNs (47–49).

Reference prediction models based on tabular data (clinical factors D1 and radiomics features D2) were constructed using the conventional machine learning methods logistic regression (M1) and random forest (M2). In addition, two deep learning approaches were also tested on these tabular data: one using a simple FCNN (M3) and the other using a FCNN with interactions between network nodes (M4) to learn possible feature interactions within the data. See Supplementary material C for details. The input data to models M1–M4 were: (i) all features in the clinical data (D1), (ii) all features in the radiomics data (D2), (iii) the combination of all tabular data (D1 + D2) and (iv) subsets of (i), (ii) or (iii) based on features selected by RENT at least once (1%) or at least 50 times (50%) out of 100 RENT runs (see Section 2.6).

Since the input images in this study were 3D images, a 3D version of the original EfficientNet was required. While constructing an EfficientNet that works with 3D images is possible in theory, a full-scaled 3D EfficientNet is still limited by computing power and suffers from the curse of dimensionality (47). Therefore, we designed a downscaled 3D version of the EfficientNet CNN to derive patterns or possibly radiomics features from 3D image input (D3) (Supplementary material C and Supplementary Table C5). With this CNN, three outcome prediction models using the following different groups of image input were evaluated: (i) PET and CT (M5); (ii) PET, CT and GTVp (M6); (iii) PET, CT, GTVp and GTVn (M7). Note that model M5 was based only on the PET/CT images and did not use ROI contours (contour-free model).

Since there were two different input data types, tabular data and image data, we wanted to investigate the benefit of combining models trained on different data types. These combinations resulted in combined models (i.e., ensembles of models) with the following input combinations: D1 + D3, D2 + D3 and D1 + D2 + D3. The primary outputs of all models M1–M7 were class probabilities within the range [0, 1]. Based on the receiver operating characteristic curve (ROC) analysis of OUS data, an optimal threshold of 0.5 for the predicted class probability was selected to separate class 0 and class 1. We combined the different models by averaging their predicted class probabilities, giving the *ensemble average*.

Model hyperparameters were optimized based on a weighted score (Supplementary material C) calculated from the validation area under the receiver operating characteristic curve (AUC), Matthew’s correlation coefficient (MCC), F1 score on both class 1 and class 0, and the training F1 score on class 1. See Section 2.8 for details. The hyperparameters that were optimized were the model regularization parameter for the logistic model M1, the number of trees (estimators) and maximum features in each split for the random forest model M2, and the loss function, dropout rate, and architecture complexity for the deep learning models M3–M7. Detailed information on the selected hyperparameters used in models M1–M7, as well as the chosen architectures of models M3–M7 are shown in Supplementary Tables C1–C5.

### 2.8. Training and evaluation metrics

**Figure 1:**
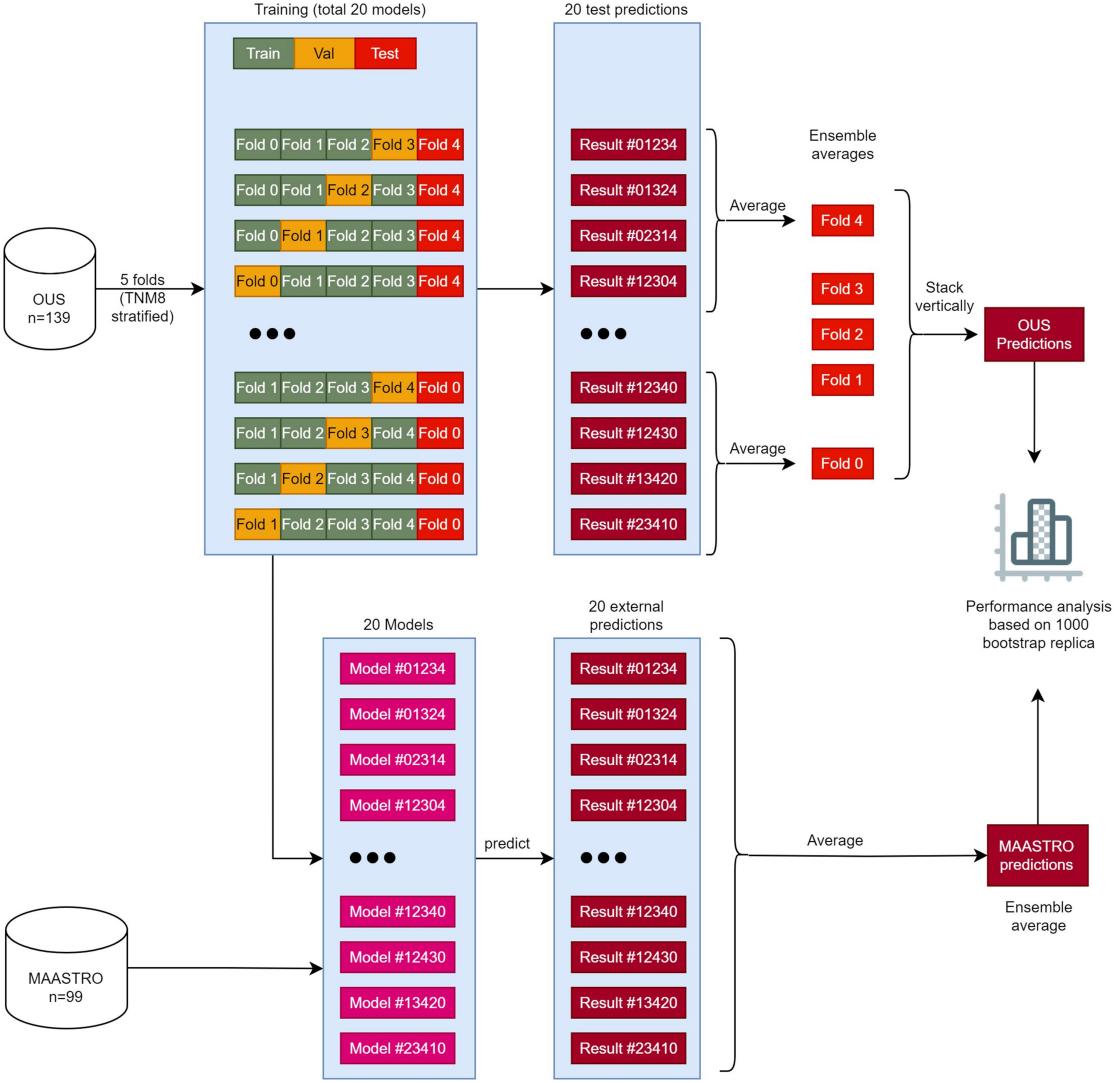
The workflow of training, validating, and testing models M1–M7. The OUS dataset was divided into five folds (top), with three folds (green) used for training, one fold (orange) used for validation and one fold (red) used for internal testing. A nested five-fold cross-validation repeated five times was then applied to these folds resulting in a total of 20 models. In this approach, four models (1–4) were trained and then tested on the internal test fold (red). The four predictions on the test fold were averaged giving the ensemble average for each patient in this fold. This process was repeated five times, creating 20 models and five ensemble averages. Then the five ensemble averages were stacked vertically, resulting in the full predictions for each patient in the OUS dataset based on the hold-out internal test data. The 20 models were then evaluated on the external MAASTRO dataset. The MAASTRO predictions for each patient were also obtained *via* ensemble averaging. To compensate for class imbalances when calculating the performance metrics, the final metrics were calculated from 1,000 bootstrap samples (patients) using 1:1 ratio between classes.

Models M1–M7 (Table 2) were first trained, validated and tested on the OUS dataset. This dataset was split into five folds, where the folds were stratified to conserve the proportion of stage I + II vs. III + IV patients (TNM8) in the full dataset. Even though a simple *K*-fold cross-validation can be used to tune model hyperparameters, we were also interested in evaluating the actual model performances (i.e., internal testing) across the OUS dataset. Therefore, we used a nested five-fold cross-validation approach (50) for hyperparameter optimization and model assessment, as shown in Figure 1, where four models were trained on three folds and validated on one fold using different train-validation fold combinations to predict each unseen test fold. This process was repeated five times, generating a total of 20 models. Since there were four different prediction outputs per patient for each test fold, we combined these outputs *via* ensemble averaging, thus generating the final OUS test prediction outputs. Note that prediction outputs for binary classification models are always within the range 0 and 1, where 0.5 was used as a threshold to decide if the prediction labels belonged to class 0 or class 1.

After training and evaluating a given model on the OUS dataset, the external MAASTRO dataset (Figure 1) was used for testing the 20 models on a different cohort, playing the role of an external test set (51). The final prediction outputs of the 20 models on the MAASTRO dataset were then averaged, giving one outcome label per patient. The full workflow of training and evaluating the models can be found in Figure 1.

Five main performance metrics emphasizing different aspects of model performance were computed: (i) Accuracy, (ii) AUC, (iii) MCC (rescaled to the interval 0 to 1), and F1 score on class 1 (iv) and class 0 (v) separately. To allow for thorough future inter-study comparisons, the following three additional performance metrics were also computed: precision, recall, and specificity. Performance metric definitions are given in the Supplementary material D.

As the event ratios (class 1 ratios) were different between the two datasets [DFS: 49% (OUS), 60% (MAASTRO); OS: 41% (OUS), 54% (MAASTRO)], all metrics were calculated from 1,000 bootstrap samples from prediction outputs of each dataset, using a 1:1 ratio between the two classes (Figure 1).

### 2.9. CNN model interpretability analysis

Even though it may be relatively straightforward to determine the input features that contribute to the outputs of conventional machine learning models such as logistic regression and random forest, this is not the case for deep learning models, especially for CNNs. The limitation in interpretability and explainability of deep learning models are often due to their non-linear characteristics and vast amount of training parameters (32). Various approaches for solving this problem provide heatmaps of important voxels that contribute to the model predictions. The main concept is based on the saliency map (52), which calculates the effect of a small change in an input voxel for the model prediction. However, saliency maps are usually noisy (53), which leads to other methods such as guided back-propagation (54), SmoothGrad (53), VarGrad (55), and GradCAM (56). In this study, we used VarGrad which has been shown to outperform other mentioned methods (57).

The VarGrad method calculates the **var**iance of the model **grad**ients based on each prediction. Let *f* be the function indicating the deep learning model where ${\mathrm{prediction}}_{\mathrm{response}}=f\left({\mathrm{input}}_{\mathrm{image}}\right)$, then the gradients *G* is the derivative of the function *f*, $G=f^{\prime }$, resulting in the saliency maps. To generate the VarGrad heatmap, we perturbed each input image by adding noise, then calculated the gradients *G* to generate the saliency map associated with the input image. We repeated these steps 20 times, and the variances of the resulting 20 different gradients for each input image provided the VarGrad heatmaps showing the important voxels associated with each input image.

For each response variable DFS and OS, we applied the VarGrad method to the highest performing CNN models M5–M7 and analyzed the resulting heatmaps to investigate which areas in the input image contributed the most to the model prediction.

## 3. Results

### 3.1. Features selected by brute-force RENT

Tables 3, 4 show the features most frequently selected by the brute-force RENT feature selection approach, as described above, when predicting DFS and OS on the OUS dataset. The complete list of features selected at least once by brute-force RENT (≥ 1% selection frequency) is shown in Supplementary material E.

**Table 3:** Features selected by RENT with at least 25% frequency for predicting DFS.

Input group	Feature	Frequency (%)
Clinical factors D1	HPV-related	98
	TNM8 stage	89
	Smoking (pack year)	41
	Tumor site—oral cavity	39
	Tumor site—oropharynx	37
Radiomics features D2	Shape feature—tumor sphericity	95
	PET texture—LBP_102	95
	Shape feature—tumor Elongation	69
	CT texture—GLSZM small area low gray level emphasis	63
	PET texture—LBP_201	48
	PET texture—GLSZM gray level non uniformity normalized	31
All tabular data. Clinical factors D1 + radiomics features D2	Shape feature—tumor sphericity	98
	Shape feature—tumor elongation	95
	PET texture—LBP_102	94
	CT texture—GLSZM small area low gray level emphasis	85
	PET texture—LBP_201	68
	HPV related	55
	PET texture—GLSZM gray level non uniformity normalized	49
	TNM8 stage	47

**Table 4:** Features selected by RENT with at least 25% frequency for predicting OS.

Input group	Feature	Frequency (%)
Clinical factors D1	TNM8 stage	100
	HPV-related	95
	Smoking (pack year)	47
	Tumor site—oropharynx	36
Radiomics features D2	Shape feature—tumor sphericity	100
	CT texture—GLCM joint average	79
	CT texture—GLCM sum average	79
	Shape feature—major axis length	57
	CT first order—maximum discrete HU	35
	PET texture—GLRLM high gray level run emphasis	34
	Shape feature—maximum tumor 3D diameter	31
	PET texture—GLCM cluster shade	25
All tabular data. Clinical factors D1 + radiomics feature D2	Shape feature—tumor sphericity	100
	TNM8 stage	88
	HPV-related	86

For input group D1 (clinical factors and standard PET parameters), seven and ten (out of 14) features were selected by RENT at least once for OS and DFS, respectively (Supplementary Tables E1, E2 and Supplementary material E). The most frequently selected features were HPV status and the TNM8 stage, followed by smoking status and the tumor site (Tables 3, 4). Out of the 374 radiomics features from D2, less than 10% were selected by RENT at least once, with the shape feature sphericity as the top feature for both outcomes. For DFS prediction, PET rather than CT texture features were more frequently selected, whereas PET and CT textures were selected about equally for OS prediction. For DFS models based on both clinical and radiomics features (D1 + D2), RENT selected 42 features where two shape features, namely sphericity and elongation, and an LBP PET texture feature were selected most frequently. Only seven clinical features were selected in this feature subset, and the remaining selected features were mostly PET texture features. Surprisingly, for the OS models based on the combined data (D1 + D2), sphericity was the only selected radiomics feature, whereas the remaining six features were clinical factors, with TMN8 stage and the HPV status being the most frequently selected after sphericity. For both outcomes, first order radiomics features and the three standard PET parameters were rarely selected by RENT (Supplementary Tables E1, E2).

### 3.2. Model performances

**Figure 2:**
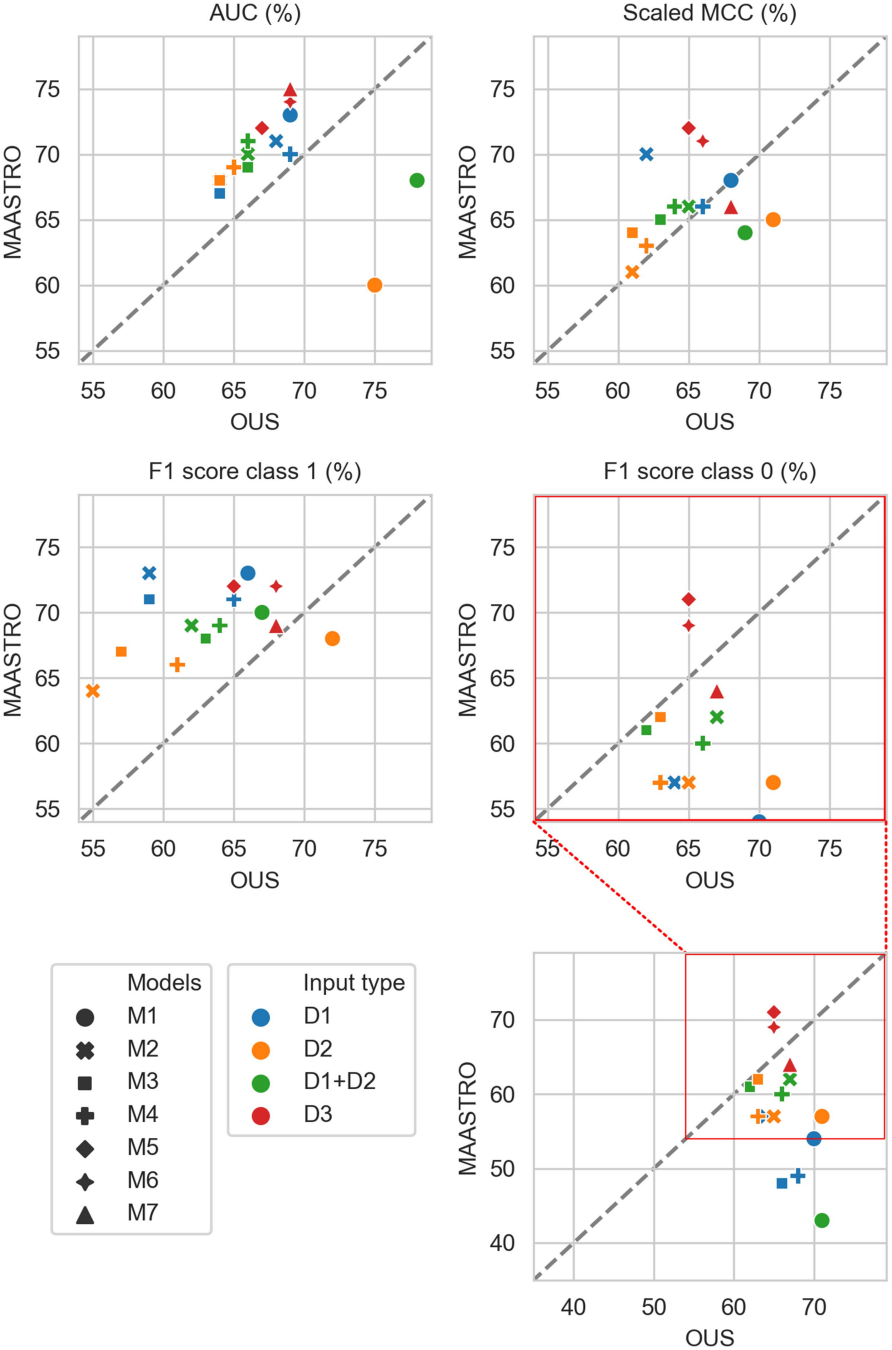
Median performance metrics for prediction of DFS by tabular based models (M1–M4) trained on all clinical data (D1, blue), all radiomics features (D2, orange) and all tabular data (D1 + D2, green), together with CNN models (D3, red) trained on PET/CT only (M5), PET/CT and GTVp (M6) and PET/CT, GTVp and GTVn (M7). All metrics were the calculated median from bootstrap sampling the OUS and MAASTRO datasets to maintain the 1:1 ratio between class 1 (event occurrence) and class 0. The *x* and *y* axes indicate model performance on OUS and MAASTRO datasets, respectively. The dashed reference line indicates equal model performance on both datasets.

**Figure 3:**
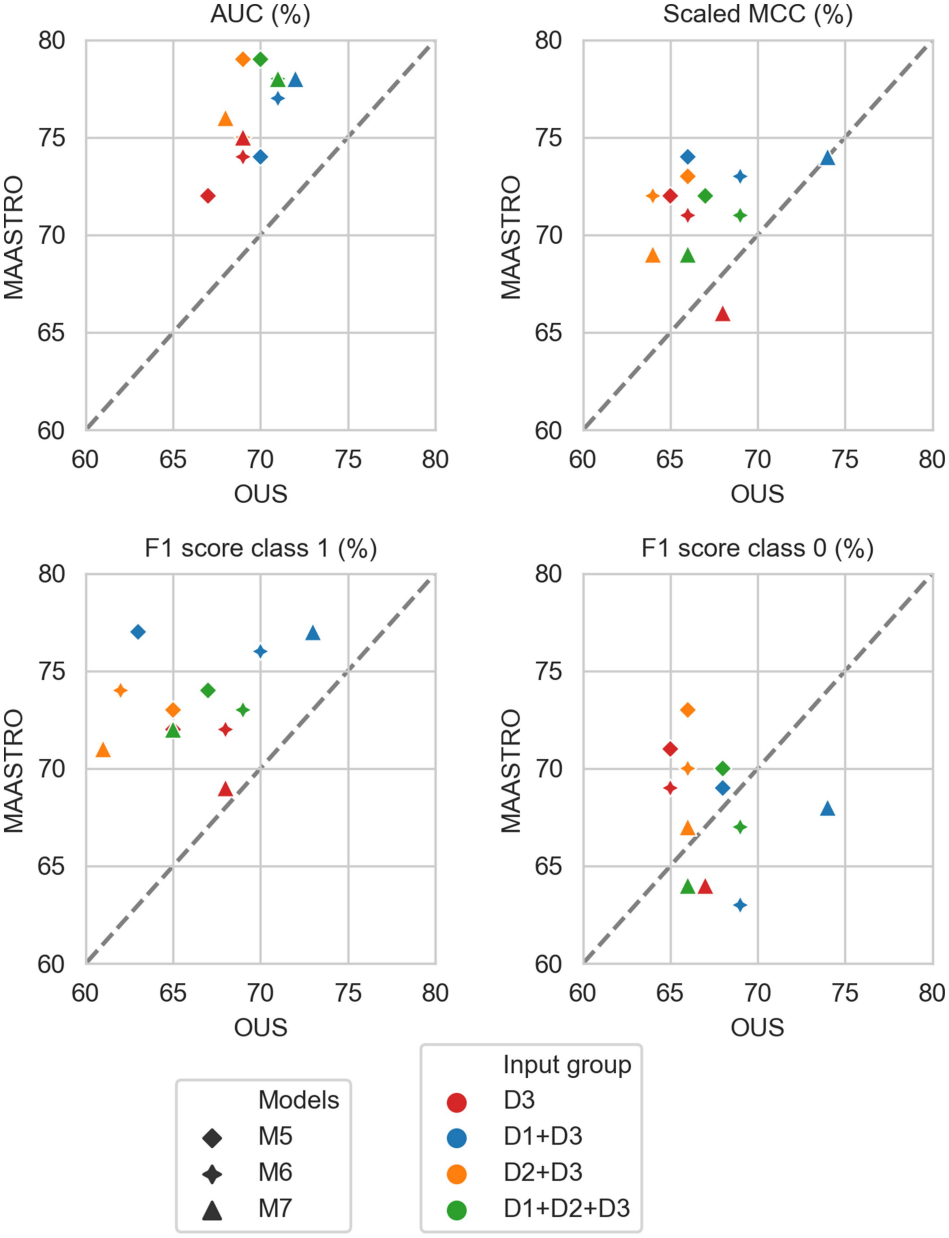
Median performance metrics for prediction of DFS by CNN models (D3, red) trained on PET/CT only (M5), PET/CT and GTVp (M6) and PET/CT, GTVp and GTVn (M7), (red) and models M5–M7 combined with tabular-based models (M1–M4) trained on clinical data (D1, blue), radiomics features (D2, orange) and all tabular data (D1 + D2, green). All metrics were the calculated median from bootstrap sampling the OUS and MAASTRO datasets to maintain the 1:1 ratio between class 1 (event occurrence) and class 0. The *x* and *y* axes indicate model performance on OUS and MAASTRO datasets, respectively. The dashed reference line indicates equal model performance on both datasets.

**Figure 4:**
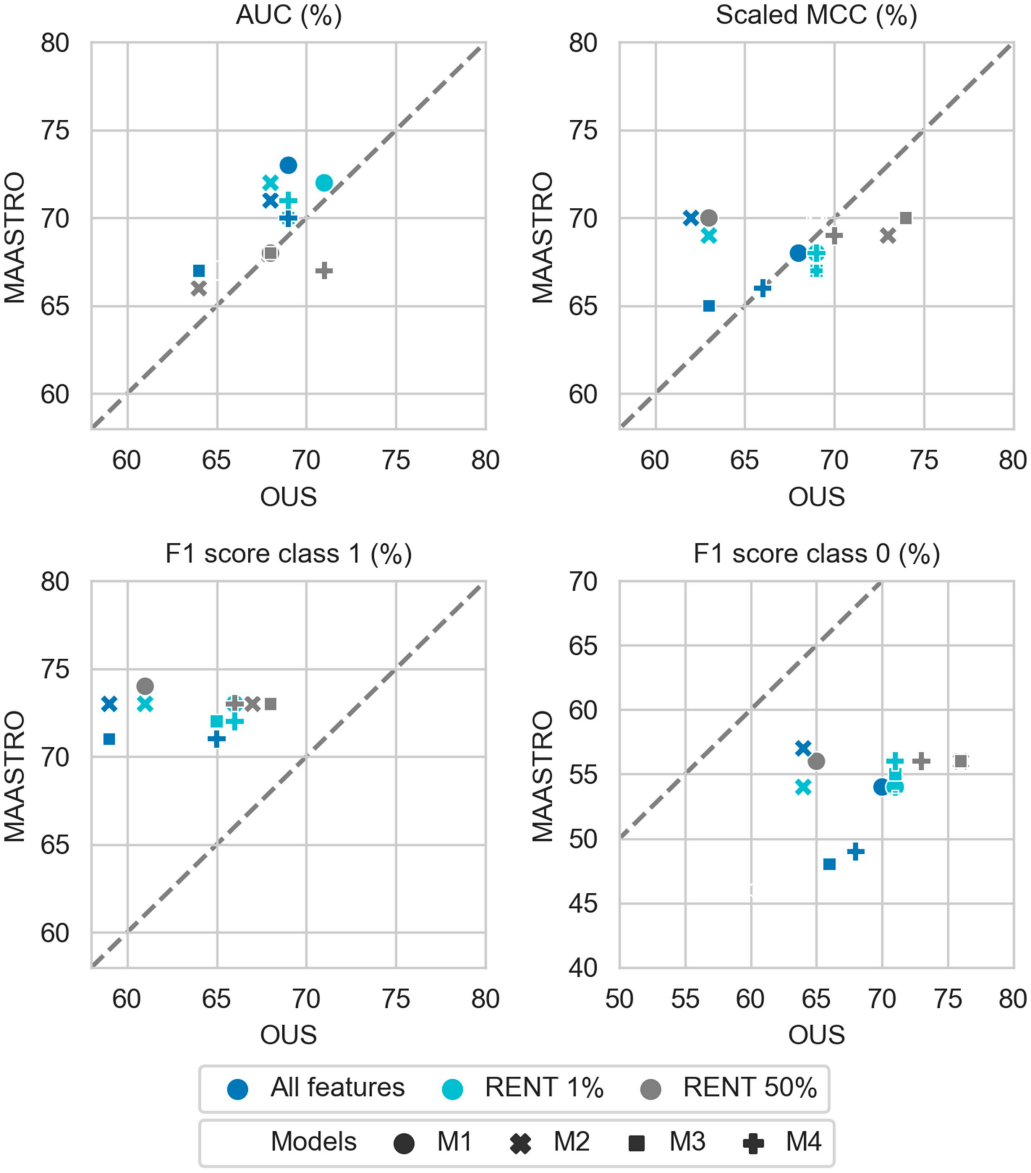
Effects of RENT feature selection on performance metrics for prediction of DFS using clinical features (D1) only by M1–M4 models trained on all clinical features D1 (dark blue), clinical features selected by RENT at least once (1%, light blue), and clinical features selected in 50% of the RENT runs (50%, gray). All metrics were the calculated median from bootstrap sampling the OUS and MAASTRO datasets to maintain the 1:1 ratio between class 1 (event occurrence) and class 0. The *x* and *y* axes indicate model performance on OUS and MAASTRO datasets, respectively. The dashed reference line indicates equal model performance on both datasets.

**Figure 5:**
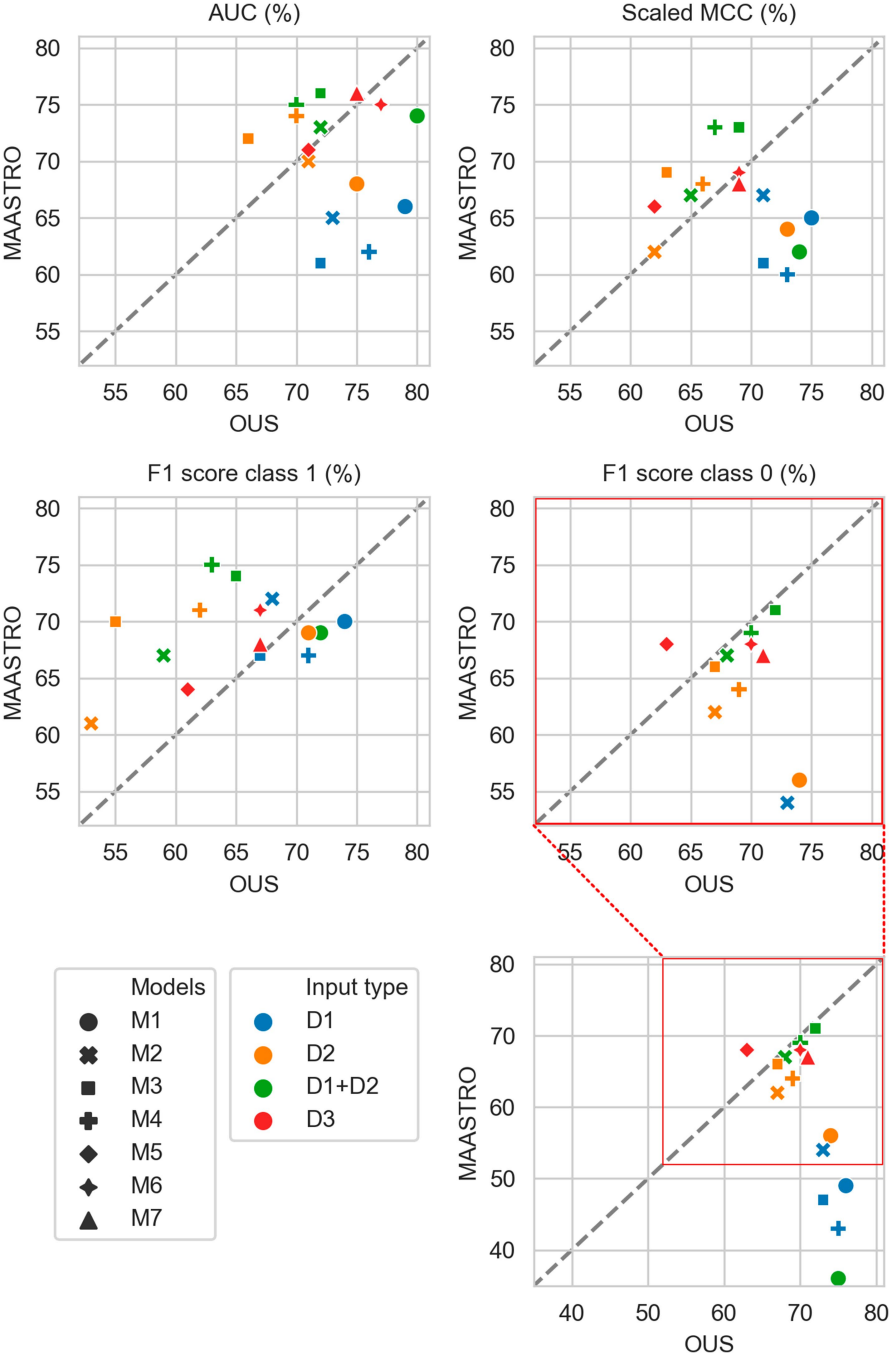
Median performance metrics for prediction of OS by tabular based models (M1–M4) trained on all clinical data (D1, blue), all radiomics features (D2, orange) and all tabular data (D1 + D2, green), together with CNN models (D3, red) trained on PET/CT only (M5), PET/CT and GTVp (M6) and PET/CT, GTVp and GTVn (M7). All metrics were the calculated median from bootstrap sampling the OUS and MAASTRO datasets to maintain the 1:1 ratio between class 1 (event occurrence) and class 0. The *x* and *y* axes indicate model performance on OUS and MAASTRO datasets, respectively. The dashed reference line indicates equal model performance on both datasets.

**Figure 6:**
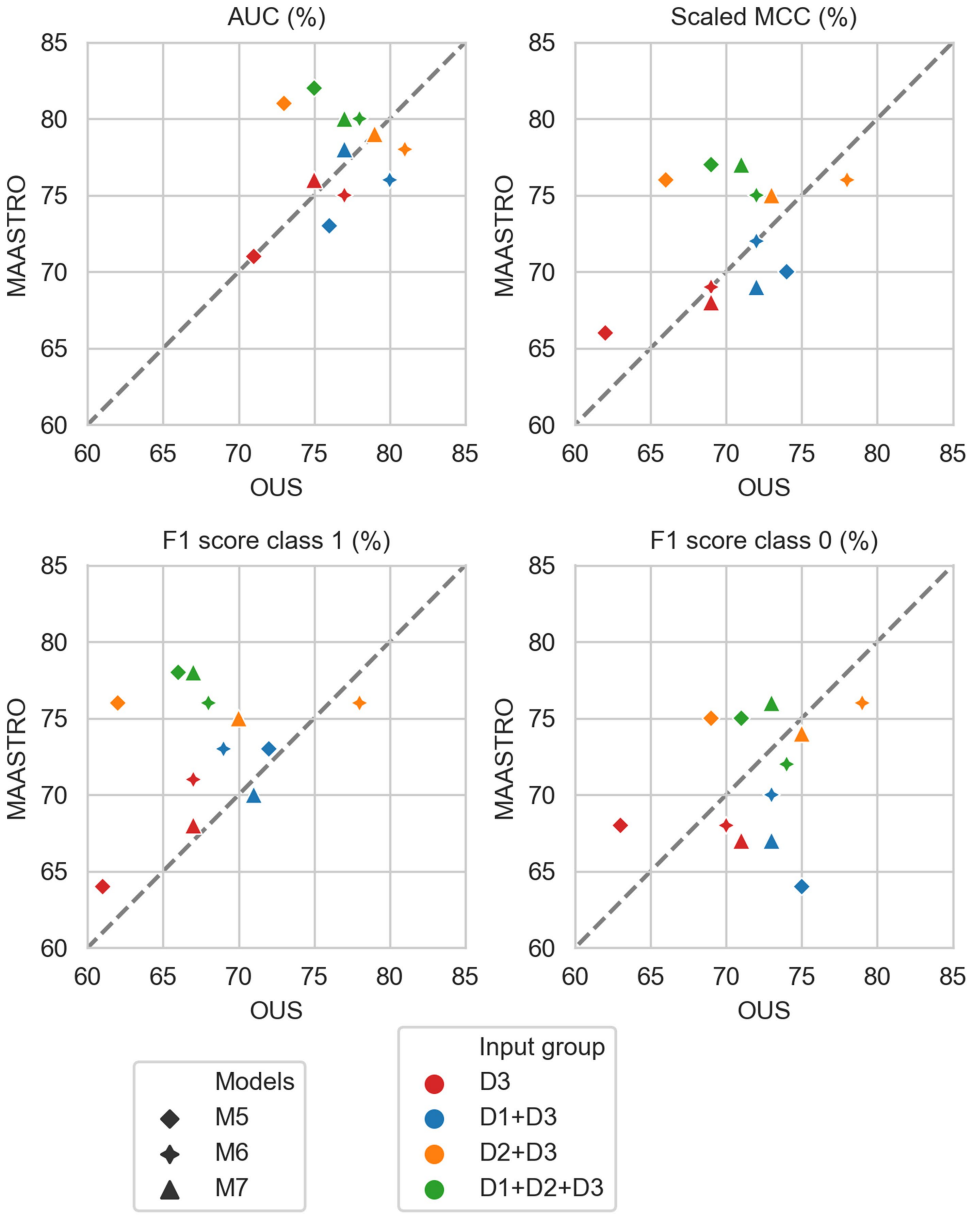
Median performance metrics for prediction of OS by CNN models (D3, red) trained on PET/CT only (M5), PET/CT and GTVp (M6) and PET/CT, GTVp and GTVn (M7) (red) and models M5–M7 combined with tabular-based models (M1–M4) trained on clinical data (D1, blue), radiomics features (D2, orange) and all tabular data (D1 + D2, green). All metrics were the calculated median from bootstrap sampling the OUS and MAASTRO datasets to maintain the 1:1 ratio between class 1 (event occurrence) and class 0. The *x* and *y* axes indicate model performance on OUS and MAASTRO datasets, respectively. The dashed reference line indicates equal model performance on both datasets.

**Figure 7:**
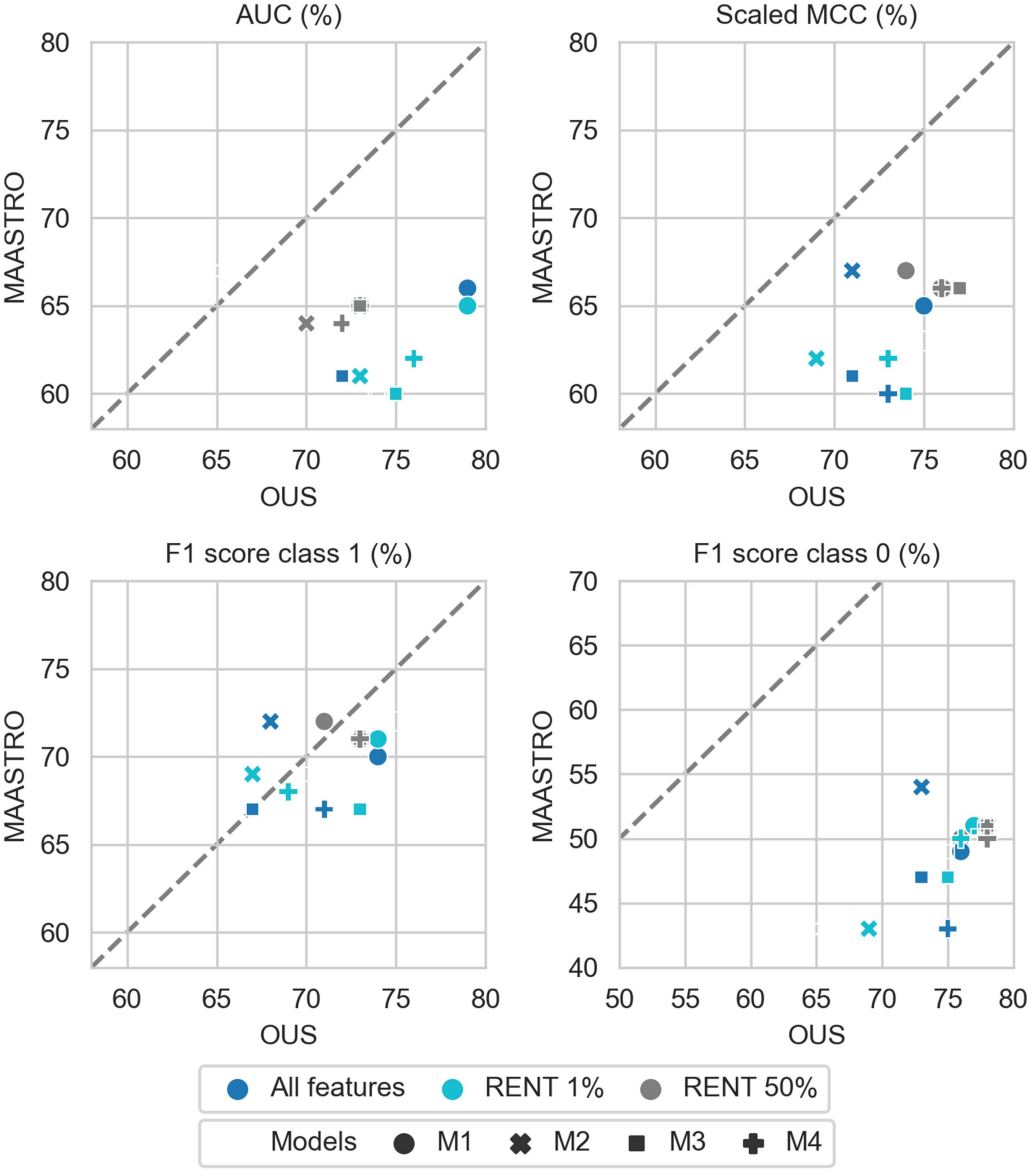
Effects of RENT feature selection on performance metrics for prediction of OS using clinical features (D1) only by M1–M4 models, trained on all clinical features D1 (dark blue), clinical features selected by RENT at least once (1%, light blue), and clinical features selected in 50% of the RENT runs (50%, gray). All metrics were the calculated median from bootstrap sampling the OUS and MAASTRO datasets to maintain the 1:1 ratio between class 1 (event occurrence) and class 0. The *x* and *y* axes indicate model performance on OUS and MAASTRO datasets, respectively. The dashed reference line indicates equal model performance on both datasets.

Figures 2–7 show the performance of DFS and OS models on the two cohorts, OUS (internal test set) and MAASTRO (external test set). A dashed reference line is given in the figures to indicate the points of equal model performance on both cohorts. Models (data points) lying along this line performed equally well on both cohorts. Data points below the diagonal dashed reference line, show models overfitting the OUS dataset whereas data points above the line show models with higher performance on the MAASTRO than the OUS set. Since the results were the median of 1,000 bootstrap samples to maintain the 1:1 ratio between class 1 (event occurrence) and class 0, the two metrics MCC (scaled) and accuracy were very similar. Thus, the accuracy is not shown in Figures 2–7. As precision, recall, and specificity were primarily included to facilitate future inter-study comparisons, they are reported in the Supplementary material only. See Supplementary Figures F1–F4 and Supplementary Tables F1, F2 for the full model performances. Note that the model standard deviations (of the mean performances on the OUS test folds; cf. Figure 1) were in the range 0.03–0.10 for all performance metrics.

#### 3.2.1. DFS prediction

Figure 2 shows that for prediction of DFS, CNN models M5–M7 were the most generalizable across the two cohorts, with high performances on both OUS and MAASTRO datasets (see also Supplementary Figure F2 and Supplementary Table F1). These CNN models, either trained only on PET and CT images (M5) without ROI contours or with additional tumor (M6) or tumor and node (M7) information, obtained similar and good performance, with AUC in the range of 67% and 75% and scaled MCC between 65% and 72% on the OUS and MAASTRO datasets. Although models M1–M4 trained on either clinical data and standard PET parameters (D1) or radiomics features (D2) obtained similar or slightly lower AUC and MCC values, they did not generalize as well on the MAASTRO dataset (Supplementary Figure F1, top and middle panels). This is evident by their class 0 F1 score being below the reference line (Figure 2, last panel). Note that the three M1 models (linear classifiers based on logistic regression) trained on any of the tabular data (D1, D2, D1 + D2) did not generalize well when considering the class 0 F1 score.

Apart from the linear logistic model M1, models M2–M4 trained on the combined clinical and radiomics features (D1 + D2) also had good generalizability across the two cohorts, but with slightly lower performance than the CNN models M5–M7 (Figure 2; Supplementary Figure F1, bottom).

Figure 3 shows CNN models M5–M7 combined with models trained on tabular data D1, D2, or D1 + D2 (see also Supplementary Figure F2). Overall, adding either clinical factors (D1) or both clinical and radiomics features (D1 + D2) to CNN models slightly improved performance with some trade-off on the class 0 F1 score (about 5%) for prediction on the external MAASTRO dataset. However, adding radiomics features only (D2) did not alter CNN model performance.

While adding more information slightly increased the performance of CNN models, removing features from the radiomics data (D2) or all tabular data [combined clinical and radiomics features (D1 + D2)] using RENT made models M1–M4 (Supplementary Figure F1, middle and bottom panels) substantially overfit to the OUS dataset, as seen by the lower performance metrics when testing on the MAASTRO set. However, as seen in Figure 4 (see also Supplementary Figure F1, top), RENT feature selection did not substantially affect models M1–M4 based on clinical features only (D1). Note that models using only two clinical features from D1 (HPV and TNM8 stage, Table 3) selected in more than 50% of the repeated RENT runs, performed similarly or even better than those trained on many more clinical features (Figure 4).

#### 3.2.2. OS prediction

As for DFS prediction, Figure 5 shows that CNN models M5–M7 predicting OS had good generalizability across the OUS and MAASTRO datasets (see also Supplementary Figure F4 and Supplementary Table F2). Models M6 and M7 with additional tumor and tumor + node information obtained similar and higher performances with AUC up to 75% and scaled MCC of almost 70% on both datasets relative to the contour-free M5 model trained only PET and CT images. Apart from the logistic model M1, tabular-based models M2–M4 trained on both clinical and radiomics features (D1 + D2), also generalized well across the two different cohorts, with similar or slightly higher performance than the CNN models (Supplementary Figure F3, bottom). Similar to the DFS predictions, the three linear logistic M1 models trained on any of the tabular data (D1, D2, D1 + D2) did not generalize well with regard to class 0 F1 score performance.

Moreover, all models M1–M4 trained on clinical data only (D1) overfitted to the OUS dataset, with all metric values below the reference line (Figure 5; Supplementary Figure F3, top panel). Models M2–M4 trained on radiomics features D2, while having the good AUC, scaled MCC and class 0 F1 score on both datasets, had large differences in the class 1 F1 score between the OUS and MAASTRO datasets (Supplementary Figure F3, middle panel).

As seen in Figure 6, combining CNN models with another model trained on tabular data (D1, D2 or D1 + D2) substantially improved model performances on both datasets, with AUC around 80% and scaled MCC from 70%–77% (Supplementary Figure F4). The highest performance on both datasets was achieved by combining CNN model M6 (PET/CT images and GTVp) and the radiomics model, evident by the combined model’s data point (Figure 6, orange four-pointed star, and Supplementary Figure F4) being close to the reference line and in the top right corner for all metrics.

As was also observed for DFS prediction, feature reduction using RENT (Supplementary Figure F3) resulted in overfitting to the OUS for models M1–M4 trained on the radiomics data (D2) or all tabular data (D1 + D2). Again, for OS models trained on clinical features only (D1), Figure 7 shows that reducing the number of clinical features to only two (HPV and TNM8 stage), did not substantially reduce model performance relative to models based on all clinical features.

### 3.3. Model interpretability analysis

**Figure 8:**
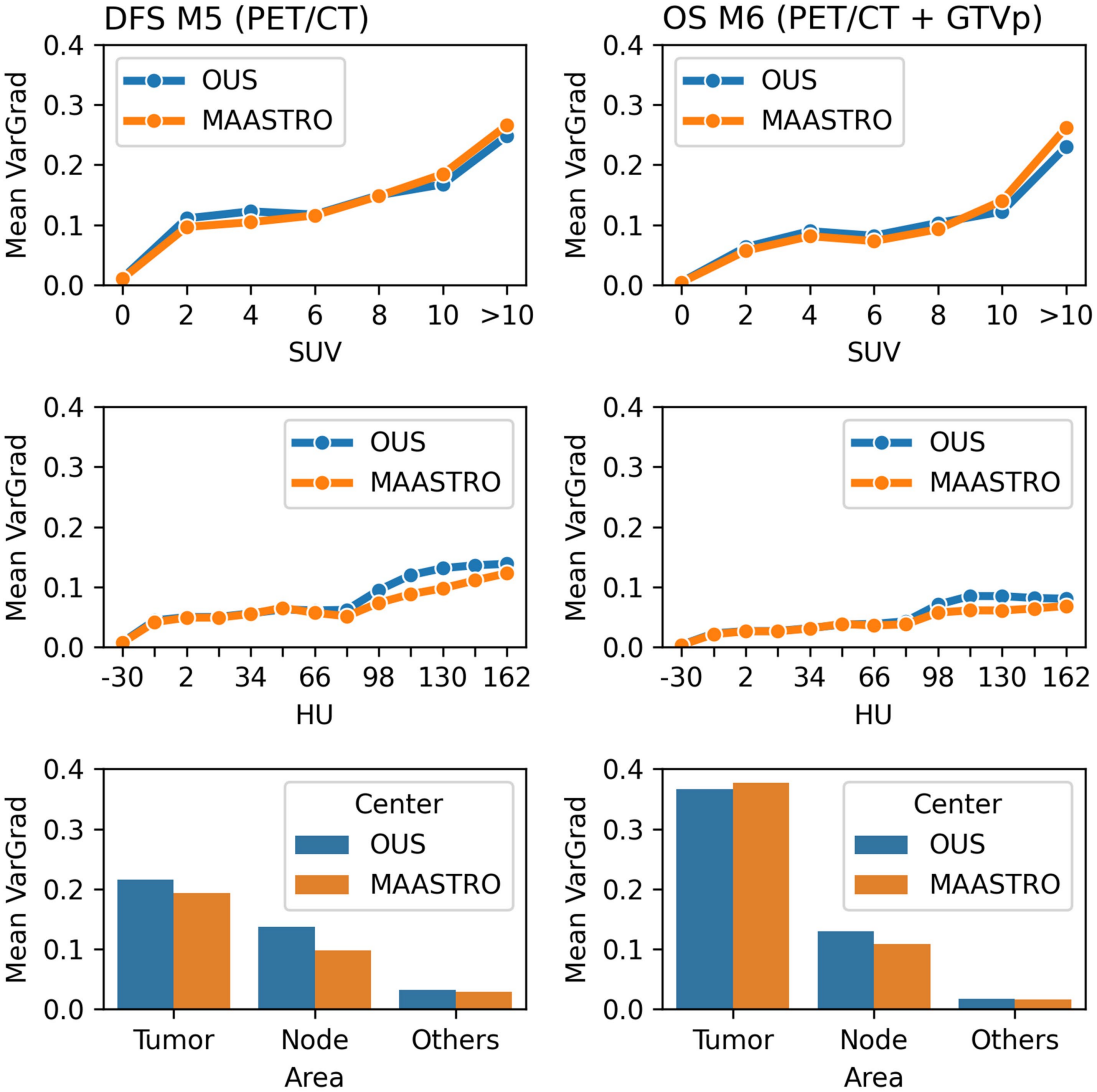
Quantitative analysis of VarGrad heatmaps based on the contour-free M5 model predicting DFS (left) and the M6 model (PET/CT + GTVp) predicting OS (right). The mean VarGrad of voxels within different areas was calculated based on SUV (first row), HU values (second row) and tumor/node/other location (last row).

Model M5 (contour-free model trained on PET and CT images only) predicting DFS and model M6 (trained on PET, CT + GTVp) predicting OS were chosen for model interpretation using the VarGrad method. Figure 8 shows the calculated mean VarGrad within different areas based on SUV values, HU values and tumor/node locations.

According to the VarGrad results, SUV played an important role in DFS and OS prediction as voxels with higher SUV had higher mean VarGrad, indicating higher contribution to the model prediction (Figure 8). Voxels with SUV between 2 and 10 had similar effect on model predictions (10%–15%), whereas voxels with SUV over 10 contributed up to 25% to the model prediction. Similarly, voxels with HU values higher than the window center (70 HU) contributed more to model predictions than those with HU values lower than the window center. However, the effect of the high HU values was not as strong as the effect of the high SUV.

The voxels within the tumor and node areas affected the model prediction more than voxels outside these areas (Figure 8). In the contour-free M5 model, the average effect of voxels within the tumor areas was double that of voxels within the node areas (20% vs. 10%). However, the average contribution of voxels within the tumor areas was almost four times higher than voxels within the node area for the M6 model (37% vs. 10%). Note that in model M5, no guided tumor or node mask was provided in the input. Model M6 used the tumor masks only (GTVp) but not the node masks.

**Figure 9:**
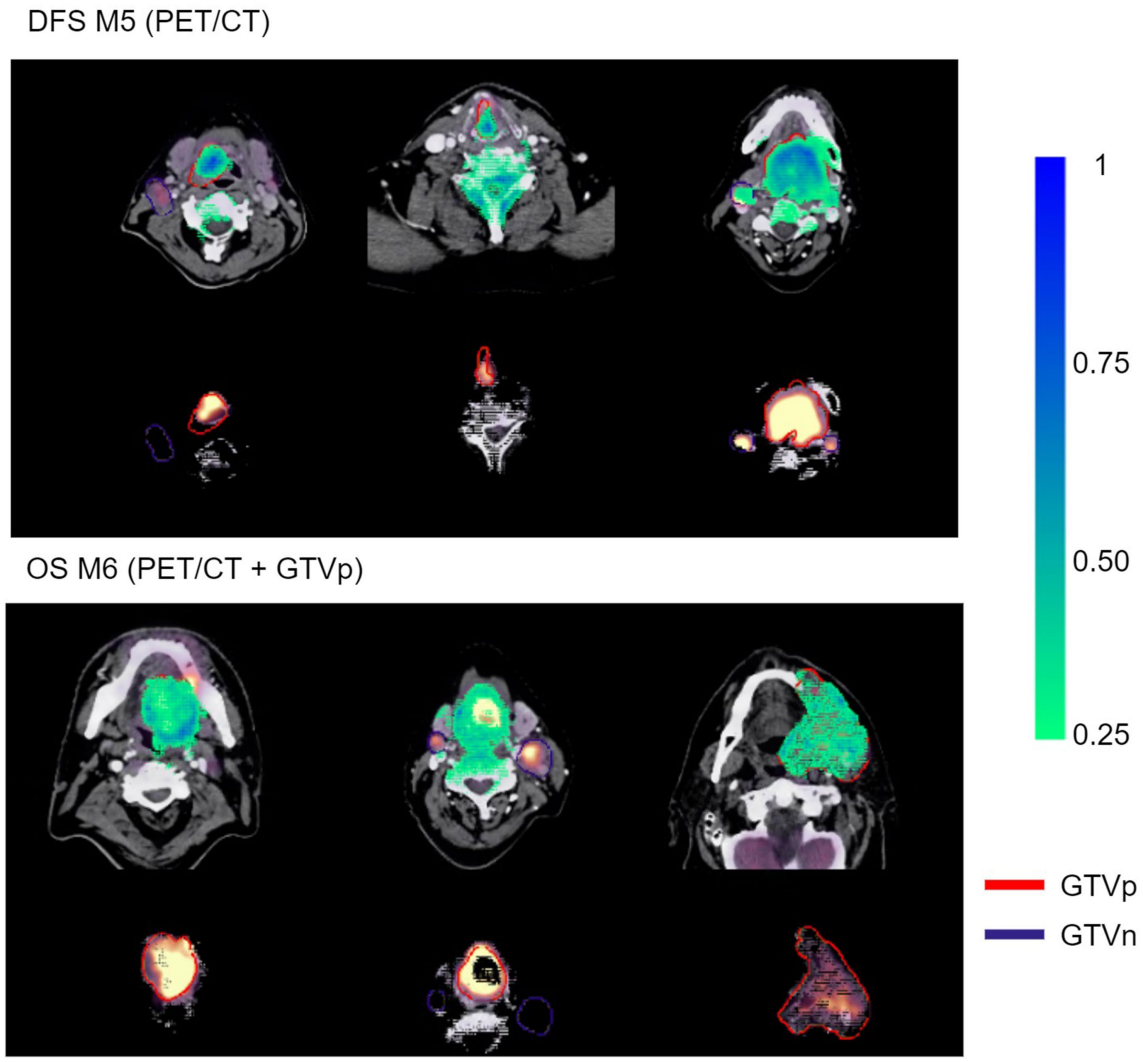
Example VarGrad heatmaps generated by the contour-free M5 model predicting DFS (top panel) and the M6 model (PET/CT + GTVp) predicting OS (bottom panel). The fused PET/CT images with the delineated primary tumor GTVp and lymph nodes GTVn are shown under the associated VarGrad heatmaps (only VarGrad values above 0.25 are shown). The parts hidden under the VarGrad heatmaps can be seen beneath the images.

Figure 9 shows examples of importance heatmaps generated by VarGrad on model M5 (trained only on PET/CT image) predicting DFS and model M6 (trained on PET/CT images and GTVp) predicting OS. In these examples, models M5 and M6 showed that the primary tumor area contributed most to model predictions, while the nodal areas GTVn were not as important. In some cases, the spine areas and regions around the tumor edges were also highlighted as important for the model predictions (Figure 9, bottom rows).

## 4. Discussion

In this study we conducted a comprehensive comparison between conventional radiomics and deep learning radiomics for prediction of OS and DFS in patients with HNC from two separate centers. Models based on clinical factors including three standard PET parameters were used as reference to assess the added benefit of radiomics. As a sanity check for our prediction models, we explored model interpretability by using a new feature selection method for tabular data (RENT) (42) as well as a state-of-the-art method for deep learning explainability (VarGrad) (55).

Our results showed that models using *all* radiomics and clinical features as well as deep learning CNN models based directly on image data with or without GTV masks performed well and could generalize to the external test cohort. Thus, conventional radiomics including clinical information and deep learning radiomics can perform similarly. This indicates that the deep learning radiomics can capture information similar to the information included in radiomics and clinical data. Interestingly, deep learning models performed well even if primary tumor and nodal contours were not provided to the models as also found in Wang et al. (15). As shown by the explainability approach, CNN models focused automatically on tumor and node regions in the images even without guidance. Based on our results, conventional radiomics appears to require complex nonlinear models for good performance as the linear logistic regression model in our case performed and generalized poorly. Models based only on either radiomics features or clinical data generally had lower performances and generalizability, suggesting that radiomics only or clinical data only is not sufficient. Thus, there was an added benefit of including radiomics with clinical factors. Feature selection of the high-dimensional radiomics data substantially reduced model generalizability, indicating that important information can be discarded by this approach.

Overall, models (excluding the linear logistic model) based on *all* clinical and radiomics features (i.e., no feature selection) as well as deep learning (CNN) models based on image data performed well on both patient cohorts with AUCs in the range 65%–75% for both outcomes. These performances are similar to those of other studies. Vallières et al. (19) obtained an AUC close to 60% for OS conventional machine learning models based on PET/CT radiomics features from both tumor and nodes, which increased significantly to about 70% when radiomics and clinical factors were combined. Likewise for OS, Liu et al. (58) obtained AUCs between 68%–90% for PET/CT radiomics of the primary tumor. Zhai et al. (23) found that for DFS, clinical and CT radiomics models performed similarly with AUCs of 65%, which increased to 70% when combined. We also observed increases when radiomics and clinical features were combined, especially for OS prediction. For OS prediction with image-based deep learning (CNN) models, concordance indices (CIs) in the range 0.60–0.78 have been reported for PET-based, CT-based and PET/CT models (15, 27, 59). Note that the HECKTOR 2021 challenge (21) reported CIs in the range of 0.67–0.71, and found that deep learning models often generalized better to the test set than conventional machine learning models for PFS, as also found in our study for DFS and OS. Submissions in HECKTOR 2022 found that combining image-based deep learning models, with radiomics and clinical features improved prediction of local regional recurrence-free survival (60). We observed a similar improvement for our combined models for OS prediction, but not for DFS.

Selection of the most important features for the tabular data (clinical and radiomics features) by the feature selector RENT (42) was intended to increase model interpretability and stability by removing redundant and non-informative features. Due to the very many radiomics features in relation to patients, feature selection is also a common step in radiomics studies (12, 16). RENT was able to significantly reduce the number of features, and conventional machine learning models based on these features obtained high performances on unseen subsets of samples derived from the internal OUS dataset. Thus, RENT was able to extract the relevant features describing the internal cohort used for feature selection and model training. However, these models did not generalize to the external test cohort. This can be seen by the decrease in the performance metrics between the internal and external datasets, particularly for the F1 score for the class 0 (patients without event), which was very low for the external set indicating many false positive predictions. It should be noted that there are considerable differences between the OUS (internal) and MAASTRO (external) cohorts with regard to factors such as HPV status (higher OUS) and tumor site as well as TNM8 stage, comorbidity and smoking which were less favorable in the MAASTRO set. Despite these differences, deep learning methods based on image data performed well on both cohorts, indicating that the CNNs, most likely due to their complexity, were able to automatically identify discriminant features across both cohorts. Though the highest prediction performance for CNN models was observed when incorporating GTV contours, models based solely on images obtained similar performances. Bypassing the need for manual contours to extract radiomics features may be an advantage in terms of simplicity and robustness. First, manual contouring is known to be time and resource-demanding. Second, substantial interobserver variability has been reported for manual contouring in a range of diagnoses including HNC (61–63). Such contour variability may impact on the derived radiomics features and is, therefore, a potential confounder in the single and multi-center radiomics studies relying on manual contours for feature extraction.

Inspection of clinical features consistently selected by RENT for the OUS cohort include HPV status, TNM8 stage, pack years and site. These correspond to factors identified as important for OS and DFS in other studies (5, 6). Vallières et al. (19) also found that the factors T-stage, N-stage, HPV status and age were significantly associated with OS. In a study of the prognostic role of clinical factors and FDG PET parameters on the same OUS cohort, Moan et al. (7) found significant associations between DFS and HPV status, comorbidity and tumor and node volume, but no significance between DFS and the PET parameters SUV_max_, MTV and TLG. Our current study using an advanced feature selection approach supports these finding as PET parameters were very rarely selected for either OS or DFS. Liu et al. (58) also found that traditional PET parameters did not predict OS or DFS as well as PET/CT radiomics features. Among the radiomics features, the shape feature sphericity, specifying the roundness of the tumor, was consistently selected for OS and DFS in our present study. This feature was also selected as a signature for OS and DFS prediction in Liu et al. (58). Interestingly, Keek et al. (20) also found tumor sphericity to be selected in a CT radiomics model for OS HNC prediction, inferring that rounder tumors had better prognosis, as also found in Apostolova et al. (64) and Aerts et al. (65). Other radiomics features highlighted by RENT include the shape features tumor major axis length and maximum 3D diameter. These two features are closely related to tumor volume, which has been identified as a treatment outcome predictor in other studies (19, 65). In addition, mainly PET and some CT texture radiomics features were selected, which are linked to intensity non-uniformity or heterogeneity within the primary tumor. Furthermore, first order radiomics features characterizing intensities and intensity distributions were seldom selected. This suggests that primary tumor heterogeneity rather than tumor intensities was more relevant for treatment outcome, supporting previous findings that tumor heterogeneity is associated with tumor aggressiveness (19, 65, 66).

Interpretability analysis of our deep learning models based on image data indicated that the CNNs focused primarily on the tumor region, followed by the nodal area and to a smaller degree on other areas. Unsurprisingly, including tumor/node contours as CNN input increased the importance of the tumor/node regions relative to other areas. Interestingly, CNN models performed similarly without inclusion of the tumor/node contours suggesting that peri-tumor environment, known to be of importance for cancer development (67), may be captured by the CNN and contribute to its prediction. The VarGrad importance score indicated that the PET signal was dominant but with some contribution from the CT signal. This is also consistent with the findings that the majority of the texture based radiomics features found in this study were derived from PET images. The dominance of the PET signal also corresponds to findings in Wang et al. (15) where CNN models based only on PET images outperformed CT-only or combined PET/CT models for prediction of OS and distant metastasis. Inspection of VarGrad importance heatmaps show that the tumor and node regions with peri-tumor surroundings were highlighted, which can be coupled to the high signal of these regions in FDG PET images. In addition, VarGrad heatmaps also in some cases included the spine and jaw, which can be linked to regions captured by CT images. One can speculate whether the CNN infers which regions are coupled to outcome, namely the tumor/nodes and surroundings, and which are not, namely the spine or jaw, and uses this information in its prediction.

This study has some limitations. It should be noted that only a moderate number of patients were included (OUS: *n* = 139; MAASTRO: *n* = 99). This was in part attributed to the fact that HPV status was not available for all (oropharyngeal cancer) patients, which in turn relates to the retrospective nature of this study. Furthermore, the included patients were not staged prospectively according to the TNM8 system, and the retrospective re-staging according to TNM8 only allowed differentiation of stage I–II vs. III–IV patients. As data were collected retrospectively, there are also differences in the treatment regimens, which could potentially impact on model performance and act as confounders. Thus, prospective studies on HNC outcome prediction, preferably with higher volume multi-centric data, would be desirable. In addition, HNC is known to be a heterogeneous group of malignancies with different characteristics and incidence rates. Analysis of one or more high-incidence and/or highly distinct HNC subgroups, such as HPV positive oropharyngeal cancers, would therefore be highly relevant. On the other hand, the mixed analysis of this study made it possible to capture general characteristics of HNC that are not dependent on primary tumor site or a particular subgroup. Moreover, a subgroup analysis would require more patients than in our present work. The aim of our present work was to conduct a comprehensive comparison of different approaches and input data. Thus, a natural extension of this study would be a more in-depth analysis of one selected model, including model fine-tuning and decision-curve analysis (68), possibly as part of the above subgroup analysis.

In summary, deep learning radiomics using image-based CNN models outperformed conventional radiomics and clinical models with regard to both performance and generalizability across cohorts from two different centers for prediction of OS and DFS in HNC. Combining these image-based models with clinical data and conventional radiomics features increased performance. Thus, image-based CNN models were able to automatically extract relevant features discriminating between patients experiencing different treatment outcomes. Interestingly, image-based CNN models trained without tumor and node contours achieved as high or nearly as high performances as models trained with contours. Thus, deep learning models based on contour-free pre-treatment images could perhaps in the future contribute to an initial screening for patients at high risk.

## Data availability statement

The data analyzed in this study is subject to the following licenses/restrictions: access to the datasets requires approval by the Ethics Committees. Requests to access these datasets should be directed to CF, cecilia.futsaether@nmbu.no.

## Ethics statement

The studies involving human participants were reviewed and approved by The Regional Ethics Committee (REC) for Medical and Health Research Ethics and Institutional Review Board (IRB). Written informed consent for participation was not required for this study in accordance with the national legislation and the institutional requirements.

## Author contributions

BH: conceptualization, data curation, formal analysis, methodology, and software for data preprocessing, data partitioning, experiments and result analysis, visualization, writing—original draft, and writing—review and editing. AG: conceptualization, data curation, formal analysis, writing—original draft, and writing—review and editing. OT and KL: conceptualization, methodology, software for feature selection, writing—original draft, and writing—review and editing. IK: conceptualization, data curation, and writing—review and editing. FH, WE, and ED: conceptualization, data curation, funding acquisition, and writing—review and editing. EM: conceptualization, data curation, funding acquisition, methodology, and writing—review and editing. CF: conceptualization, data curation, funding acquisition, methodology, supervision, project administration, writing—original draft, and writing—review and editing. All authors contributed to the article and approved the submitted version.

## Funding

The collection of the OUS dataset used in the present study was conducted as part of work supported by the Norwegian Cancer Society (grant numbers 160907-2014 and 182672-2016).

## Conflict of interest

The authors declare that the research was conducted in the absence of any commercial or financial relationships that could be construed as a potential conflict of interest.

## Publisher’s note

All claims expressed in this article are solely those of the authors and do not necessarily represent those of their affiliated organizations, or those of the publisher, the editors and the reviewers. Any product that may be evaluated in this article, or claim that may be made by its manufacturer, is not guaranteed or endorsed by the publisher.

## Footnotes

^1^Available at: https://github.com/NMBU-Data-Science/imskaper.

## References

[1] Ferlay J Colombet M Soerjomataram I Dyba T Randi G Bettio M. Cancer incidence and mortality patterns in Europe: estimates for 40 countries and 25 major cancers in 2018. Eur J Cancer. (2018) 103:356–87. doi: 10.1016/j.ejca.2018.07.005, PMID: 30100160

[2] Haddad RI Shin DM. Recent advances in head and neck cancer. N Engl J Med. (2008) 359:1143–54. doi: 10.1056/NEJMra0707975 18784104

[3] Argiris A Karamouzis MV Raben D Ferris RL. Head and neck cancer. Lancet. (2008) 371:1695–709. doi: 10.1016/S0140-6736(08)60728-X, PMID: 18486742 PMC7720415

[4] Halperin EB Wazer DE Perez CA Brady LW. Perez & Brady’s principles and practice of radiation oncology. Philadelphia: Wolters Kluwer Health (2013).

[5] Pfister DG Spencer S Adelstein D Adkins D Anzai Y Brizel DM. Head and neck cancers, version 2.2020, NCCN clinical practice guidelines in oncology. J Natl Compr Cancer Netw. (2020) 18:873–98. doi: 10.6004/jnccn.2020.0031, PMID: 32634781

[6] Lechner M Liu J Masterson L Fenton TR. HPV-associated oropharyngeal cancer: epidemiology, molecular biology and clinical management. Nat Rev Clin Oncol. (2022) 19:306–27. doi: 10.1038/s41571-022-00603-7, PMID: 35105976 PMC8805140

[7] Moan JM Amdal CD Malinen E Svestad JG Bogsrud TV Dale E. The prognostic role of ^18^F-fluorodeoxyglucose PET in head and neck cancer depends on HPV status. Radiother Oncol. (2019) 140:54–61. doi: 10.1016/j.radonc.2019.05.019, PMID: 31177043

[8] Schouten CS Hakim S Boellaard R Bloemena E Doornaert PA Witte BI. Interaction of quantitative ^18^F-FDG-PET-CT imaging parameters and human papillomavirus status in oropharyngeal squamous cell carcinoma. Head Neck. (2016) 38:529–35. doi: 10.1002/hed.23920, PMID: 25352335

[9] Afshar P Mohammadi A Plataniotis KN Oikonomou A Benali H. From handcrafted to deep-learning-based cancer radiomics: challenges and opportunities. IEEE Signal Process Mag. (2019) 36:132–60. doi: 10.1109/MSP.2019.2900993 PMC6753949 31543691

[10] Bogowicz M Riesterer O Ikenberg K Stieb S Moch H Studer G. Computed tomography radiomics predicts HPV status and local tumor control after definitive radiochemotherapy in head and neck squamous cell carcinoma. Int J Radiat Oncol. (2017) 99:921–8. doi: 10.1016/j.ijrobp.2017.06.002, PMID: 28807534

[11] Tortora M Gemini L Scaravilli A Ugga L Ponsiglione A Stanzione A. Radiomics applications in head and neck tumor imaging: a narrative review. Cancers. (2023) 15:1174. doi: 10.3390/cancers15041174, PMID: 36831517 PMC9954362

[12] Zwanenburg A. Radiomics in nuclear medicine: robustness, reproducibility, standardization, and how to avoid data analysis traps and replication crisis. Eur J Nucl Med Mol Imaging. (2019) 46:2638–55. doi: 10.1007/s00259-019-04391-8, PMID: 31240330

[13] Lambin P Leijenaar RTH Deist TM Peerlings J de Jong EEC van Timmeren J. Radiomics: the bridge between medical imaging and personalized medicine. Nat Rev Clin Oncol. (2017) 14:749–62. doi: 10.1038/nrclinonc.2017.141, PMID: 28975929

[14] Lambin P Rios-Velazquez E Leijenaar R Carvalho S van Stiphout RGPM Granton P. Radiomics: extracting more information from medical images using advanced feature analysis. Eur J Cancer. (2012) 48:441–6. doi: 10.1016/j.ejca.2011.11.036, PMID: 22257792 PMC4533986

[15] Wang Y Lombardo E Avanzo M Zschaek S Weingärtner J Holzgreve A. Deep learning based time-to-event analysis with PET, CT and joint PET/CT for head and neck cancer prognosis. Comput Methods Prog Biomed. (2022) 222:106948. doi: 10.1016/j.cmpb.2022.106948, PMID: 35752119

[16] Parmar C Grossmann P Rietveld D Rietbergen MM Lambin P Aerts HJWL. Radiomic machine-learning classifiers for prognostic biomarkers of head and neck cancer. Front Oncol. (2015) 5:5. doi: 10.3389/fonc.2015.00272 26697407 PMC4668290

[17] Parmar C Grossmann P Bussink J Lambin P Aerts HJWL. Machine learning methods for quantitative radiomic biomarkers. Sci Rep. (2015) 5:13087. doi: 10.1038/srep13087, PMID: 26278466 PMC4538374

[18] Zhai T-T Langendijk JA van Dijk LV Halmos GB Witjes MJH Oosting SF. The prognostic value of CT-based image-biomarkers for head and neck cancer patients treated with definitive (chemo-)radiation. Oral Oncol. (2019) 95:178–86. doi: 10.1016/j.oraloncology.2019.06.020, PMID: 31345388

[19] Vallières M Kay-Rivest E Perrin LJ Liem X Furstoss C Aerts HJWL. Radiomics strategies for risk assessment of tumour failure in head-and-neck cancer. Sci Rep. (2017) 7:10117. doi: 10.1038/s41598-017-10371-5 28860628 PMC5579274

[20] Keek SA Wesseling FWR Woodruff HC van Timmeren JE Nauta IH Hoffmann TK. A prospectively validated prognostic model for patients with locally advanced squamous cell carcinoma of the head and neck based on Radiomics of computed tomography images. Cancers. (2021) 13:3271. doi: 10.3390/cancers13133271, PMID: 34210048 PMC8269129

[21] Andrearczyk V Oreiller V Boughdad S Rest CCL Elhalawani H Jreige M. Overview of the HECKTOR Challenge at MICCAI (2022). Overview of the hecktor challenge at miccai 2021: Automatic head and neck tumor segmentation and outcome prediction in pet/ct images. In: Andrearczyk V Oreiller V Hatt M Depeursinge A (editors), Head and Neck Tumor Segmentation and Outcome Prediction. Lecture Notes in Computer Science (Cham: Springer International Publishing) (2022), 1–37.

[22] Goncalves M Gsaxner C Ferreira A Li J Puladi B Kleesiek J. Radiomics in head and neck cancer outcome predictions. Diagnostics. (2022) 12:2733. doi: 10.3390/diagnostics12112733 36359576 PMC9689406

[23] Zhai T-T Wesseling F Langendijk JA Shi Z Kalendralis P van Dijk LV. External validation of nodal failure prediction models including radiomics in head and neck cancer. Oral Oncol. (2021) 112:105083. doi: 10.1016/j.oraloncology.2020.105083, PMID: 33189001

[24] Zhai T-T Langendijk JA van Dijk LV van der Schaaf A Sommers L Vemer-van den Hoek JGM. Pre-treatment radiomic features predict individual lymph node failure for head and neck cancer patients. Radiother Oncol. (2020) 146:58–65. doi: 10.1016/j.radonc.2020.02.005, PMID: 32114267

[25] van Dijk LV Langendijk JA Zhai T-T Vedelaar TA Noordzij W Steenbakkers RJHM. Delta-radiomics features during radiotherapy improve the prediction of late xerostomia. Sci Rep. (2019) 9:12483. doi: 10.1038/s41598-019-48184-3 31462719 PMC6713775

[26] Bogowicz M Riesterer O Stark LS Studer G Unkelbach J Guckenberger M. Comparison of PET and CT radiomics for prediction of local tumor control in head and neck squamous cell carcinoma. Acta Oncol. (2017) 56:1531–6. doi: 10.1080/0284186X.2017.1346382, PMID: 28820287

[27] Diamant A Chatterjee A Vallières M Shenouda G Seuntjens J. Deep learning in head & neck cancer outcome prediction. Sci Rep. (2019) 9:2764. doi: 10.1038/s41598-019-39206-1, PMID: 30809047 PMC6391436

[28] Yip SSF Aerts HJWL. Applications and limitations of radiomics. Phys Med Biol. (2016) 61:R150–66. doi: 10.1088/0031-9155/61/13/R150, PMID: 27269645 PMC4927328

[29] Zwanenburg A Vallières M Abdalah MA Aerts HJWL Andrearczyk V Apte A. The image biomarker standardization initiative: standardized quantitative radiomics for high-throughput image-based phenotyping. Radiology. (2020) 295:328–38. doi: 10.1148/radiol.2020191145, PMID: 32154773 PMC7193906

[30] Reyes M Meier R Pereira S Silva CA Dahlweid FM von Tengg-Kobligk H. On the interpretability of artificial intelligence in radiology: challenges and opportunities. Radiol Artif Intell. (2020) 2:e190043. doi: 10.1148/ryai.2020190043 32510054 PMC7259808

[31] Zhang X Zhang Y Zhang G Qiu X Tan W Yin X. Deep learning with radiomics for disease diagnosis and treatment: challenges and potential. Front Oncol. (2022) 12:12. doi: 10.3389/fonc.2022.773840 PMC8891653 35251962

[32] Suarez OD Bach S Binder A Montavon G Klauschen F Müller K-R. On pixel-wise explanations for non-linear classifier decisions by layer-wise relevance propagation. PLoS One. (2015) 10:e0130140. doi: 10.1371/journal.pone.0130140 26161953 PMC4498753

[33] Cho H-h Lee HY Kim E Lee G Kim J Kwon J. Radiomics-guided deep neural networks stratify lung adenocarcinoma prognosis from CT scans. Commun Biol. (2021) 4:1286. doi: 10.1038/s42003-021-02814-7, PMID: 34773070 PMC8590002

[34] Groendahl AR Skjei Knudtsen I Huynh BN Mulstad M Moe YM Knuth F. A comparison of methods for fully automatic segmentation of tumors and involved nodes in PET/CT of head and neck cancers. Phys Med Biol. (2021) 66:065012. doi: 10.1088/1361-6560/abe553, PMID: 33666176

[35] Moe YM Groendahl AR Tomic O Dale E Malinen E Futsaether CM. Deep learning-based auto-delineation of gross tumour volumes and involved nodes in PET/CT images of head and neck cancer patients. Eur J Nucl Med Mol Imaging. (2021) 48:2782–92. doi: 10.1007/s00259-020-05125-x, PMID: 33559711 PMC8263429

[36] Groendahl AR Huynh BN Tomic O Sovik A Dale E Malinen E. Automatic gross tumor segmentation of canine head and neck cancer using deep learning and cross-species transfer learning. Front Vet Sci. (2023) 10:1143986. doi: 10.3389/fvets.2023.1143986 37026102 PMC10070749

[37] Adeoye J Hui LL Su YX. Data-centric artificial intelligence in oncology: a systematic review assessing data quality in machine learning models for head and neck cancer. J Big Data. (2023) 10:1–25. doi: 10.1186/s40537-023-00703-w36618886

[38] Lydiatt WM Patel SG O’Sullivan B Brandwein MS Ridge JA Migliacci JC. Head and neck cancers-major changes in the American Joint Committee on cancer eighth edition cancer staging manual. CA Cancer J Clin. (2017) 67:122–37. doi: 10.3322/caac.21389, PMID: 28128848

[39] DAHANCA. Radiotherapy guidelines 2013. Aarhus: Danish Head and Neck Cancer Group (DAHANCA) (2013).

[40] van Griethuysen JJM Fedorov A Parmar C Hosny A Aucoin N Narayan V. Computational radiomics system to decode the radiographic phenotype. Cancer Res. (2017) 77:e104–7. doi: 10.1158/0008-5472.CAN-17-0339, PMID: 29092951 PMC5672828

[41] Montagne C Kodewitz A Vigneron V Giraud V Lelandais S. 3D local binary pattern for PET image classification by SVM, application to early Alzheimer disease diagnosis. 6th International Conference on Bio-Inspired Systems and Signal Processing (BIOSIGNALS 2013); (2013).

[42] Jenul A Schrunner S Liland KH Indahl UG Futsaether CM Tomic O. RENT-repeated elastic net technique for feature selection. IEEE Access. (2021) 9:152333–46. doi: 10.1109/ACCESS.2021.3126429

[43] Jenul A Schrunner S Huynh BN Tomic O. RENT: a Python package for repeated elastic net feature selection. J Open Source Softw. (2021) 6:3323. doi: 10.21105/joss.03323

[44] Wong LM Ai QYH Zhang R Mo F King AD. Radiomics for discrimination between early-stage nasopharyngeal carcinoma and benign hyperplasia with stable feature selection on MRI. Cancers. (2022) 14:3433. doi: 10.3390/cancers14143433, PMID: 35884494 PMC9324280

[45] Studer E Schönecker L Meylan M Stucki D Dijkman R Holwerda M. Prevalence of BRD-related viral pathogens in the upper respiratory tract of Swiss veal calves. Animals. (2021) 11:1940. doi: 10.3390/ani11071940, PMID: 34209718 PMC8300226

[46] Kunhoth J Al-Maadeed S Kunhoth S Akbari Y. Automated systems for diagnosis of dysgraphia in children: a survey and novel framework2022, (2022). *arXiv* [Epub ahead of preprint]. Available at: 10.48550/arXiv.2206.13043.

[47] Berisha V Krantsevich C Hahn PR Hahn S Dasarathy G Turaga P. Digital medicine and the curse of dimensionality. NPJ Digit Med. (2021) 4:153. doi: 10.1038/s41746-021-00521-5, PMID: 34711924 PMC8553745

[48] Tan MX Le QV. EfficientNet: rethinking model scaling for convolutional neural networks. Pr Mach Learn Res. (2019):97. doi: 10.48550/arXiv.1905.11946

[49] Yang Y Zhang L Du M Bo J Liu H Ren L. A comparative analysis of eleven neural networks architectures for small datasets of lung images of COVID-19 patients toward improved clinical decisions. Comput Biol Med. (2021) 139:104887. doi: 10.1016/j.compbiomed.2021.104887, PMID: 34688974 PMC8461289

[50] Cawley GC Talbot NLC. On over-fitting in model selection and subsequent selection bias in performance evaluation. J Mach Learn Res. (2010) 11:2079–107. doi: 10.5555/1756006.1859921

[51] Kleppe A Skrede OJ De Raedt S Liestol K Kerr DJ Danielsen HE. Designing deep learning studies in cancer diagnostics. Nat Rev Cancer. (2021) 21:199–211. doi: 10.1038/s41568-020-00327-9 33514930

[52] Simonyan K Zisserman A. Very deep convolutional networks for large-scale image recognition. *arXiv*. [Epub ahead of preprint]. Available at: 10.48550/arXiv.1409.1556 (2014).

[53] Smilkov D Thorat N Kim B Viégas F Wattenberg M. Smoothgrad: removing noise by adding noise. *arXiv*. [Epub ahead of preprint]. Available at: 10.48550/arXiv.1706.03825. (2017).

[54] Springenberg JT Dosovitskiy A Brox T Riedmiller M. Striving for simplicity: the all convolutional net. *arXiv*. [Epub ahead of preprint]. Available at: 10.48550/arXiv.1412.6806. (2014).

[55] Adebayo J Gilmer J Muelly M Goodfellow I Hardt M Kim B. Sanity checks for saliency maps. *arXiv*. [Epub ahead of preprint]. Available at: 10.48550/arXiv.1810.03292. (2018).

[56] Selvaraju RR Cogswell M Das A Vedantam R Parikh D Batra D. Grad-CAM: visual explanations from deep networks via gradient-based localization. (2017) IEEE International Conference on Computer Vision (ICCV) 2017. 618–626.

[57] Hooker S Erhan D Kindermans PJ Kim B. A benchmark for interpretability methods in deep neural networks. Advances in Neural Information Processing Systems 32 (NIPS 2019). (2019); 32.

[58] Liu Z Cao Y Diao W Cheng Y Jia Z Peng X. Radiomics-based prediction of survival in patients with head and neck squamous cell carcinoma based on pre- and post-treatment 18F-PET/CT. Aging. (2020) 12:14593–619. doi: 10.18632/aging.103508, PMID: 32674074 PMC7425452

[59] Cheng N-M Yao J Cai J Ye X Zhao S Zhao K. Deep learning for fully automated prediction of overall survival in patients with oropharyngeal Cancer using FDG-PET imaging. Clin Cancer Res. (2021) 27:3948–59. doi: 10.1158/1078-0432.CCR-20-4935 33947697

[60] Meng M Bi L Feng D Kim J. Radiomics-enhanced deep multi-task learning for outcome prediction in head and neck Cancer. In: Andrearczyk V Oreiller V Hatt M Depeursinge A, (editors), Head and neck tumor segmentation and outcome prediction. Lecture Notes in Computer Science. (Cham: Springer Nature Switzerland) (2023). 135–143.

[61] Bird D Scarsbrook AF Sykes J Ramasamy S Subesinghe M Carey B. Multimodality imaging with CT, MR and FDG-PET for radiotherapy target volume delineation in oropharyngeal squamous cell carcinoma. BMC Cancer. (2015) 15:15. doi: 10.1186/s12885-015-1867-8 26530182 PMC4632362

[62] Gudi S Ghosh-Laskar S Agarwal JP Chaudhari S Rangarajan V Nojin Paul S. Interobserver variability in the delineation of gross tumour volume and specified organs-at-risk during IMRT for head and neck cancers and the impact of FDG-PET/CT on such variability at the primary site. J Med Imaging Radiat Sci. (2017) 48:184–92. doi: 10.1016/j.jmir.2016.11.003, PMID: 31047367

[63] Segedin B Petric P. Uncertainties in target volume delineation in radiotherapy - are they relevant and what can we do about them? Radiol Oncol. (2016) 50:254–62. doi: 10.1515/raon-2016-0023, PMID: 27679540 PMC5024655

[64] Apostolova I Steffen IG Wedel F Lougovski A Marnitz S Derlin T. Asphericity of pretherapeutic tumour FDG uptake provides independent prognostic value in head-and-neck cancer. Eur Radiol. (2014) 24:2077–87. doi: 10.1007/s00330-014-3269-8 24965509

[65] Aerts HJ Velazquez ER Leijenaar RT Parmar C Grossmann P Carvalho S. Decoding tumour phenotype by noninvasive imaging using a quantitative radiomics approach. Nat Commun. (2014) 5:4006. doi: 10.1038/ncomms5006, PMID: 24892406 PMC4059926

[66] Marusyk A Janiszewska M Polyak K. Intratumor heterogeneity: the Rosetta stone of therapy resistance. Cancer Cell. (2020) 37:471–84. doi: 10.1016/j.ccell.2020.03.007, PMID: 32289271 PMC7181408

[67] Wang X Chen K Wang W Li Q Liu K Li Q. Can peritumoral regions increase the efficiency of machine-learning prediction of pathological invasiveness in lung adenocarcinoma manifesting as ground-glass nodules? J Thorac Dis. (2021) 13:1327–37. doi: 10.21037/jtd-20-2981, PMID: 33841926 PMC8024795

[68] Vickers AJ Elkin EB. Decision curve analysis: a novel method for evaluating prediction models. Med Decis Mak. (2006) 26:565–74. doi: 10.1177/0272989X06295361, PMID: 17099194 PMC2577036

