# Corrigendum: Head and neck cancer treatment outcome prediction: a comparison between machine learning with conventional radiomics features and deep learning radiomics

**DOI:** 10.3389/fmed.2024.1421603

**Published:** 2024-05-14

**Authors:** Bao Ngoc Huynh, Aurora Rosvoll Groendahl, Oliver Tomic, Kristian Hovde Liland, Ingerid Skjei Knudtsen, Frank Hoebers, Wouter van Elmpt, Eirik Malinen, Einar Dale, Cecilia Marie Futsaether

**Affiliations:** ^1^Faculty of Science and Technology, Norwegian University of Life Sciences, Ås, Norway; ^2^Department of Circulation and Medical Imaging, Norwegian University of Science and Technology, Trondheim, Norway; ^3^Department of Medical Physics, Oslo University Hospital, Oslo, Norway; ^4^Department of Radiation Oncology (MAASTRO), Maastricht University Medical Center, Maastricht, Netherlands; ^5^GROW School for Oncology and Reproduction, Maastricht University Medical Center, Maastricht, Netherlands; ^6^Department of Physics, University of Oslo, Oslo, Norway; ^7^Department of Oncology, Oslo University Hospital, Oslo, Norway

**Keywords:** machine learning, deep learning, artificial intelligence, feature selection, radiomics, head and neck cancer, interpretability, outcome prediction

In the published article, there was an error in Supplementary Tables F1, F2. Incorrect numbers were inserted into the two specificity columns. All other columns are correct.

The corrected Supplementary Tables F1, F2 has been published in the original article.

The authors apologize for these errors and state that this does not change the scientific conclusions of the article in any way and does not change the original article in any way. The original Supplementary material file has been updated.

