# Supplementary material for: Head and neck cancer treatment outcome prediction: a comparison between machine learning with conventional radiomics features and deep learning radiomics
Source: Front Med (Lausanne). 2023 Aug 30;10:1217037. doi: 10.3389/fmed.2023.1217037 (PMC10498924; doi:10.3389/fmed.2023.1217037)
Supplement: Supplementary file 1 [file Data_Sheet_1.pdf]

Supplementary Material

Head and neck cancer treatment outcome prediction: A comparison between machine learning with conventional radiomics features and deep learning radiomics

Bao Ngoc Huynh[1], Aurora Rosvoll Groendahl[1], Oliver Tomic[1], Kristian Hovde Liland[1], Ingerid Skjei Knudtsen[2,3], Frank Hoebers[4,5], Wouter van Elmpt[4,5], Eirik Malinen[3,6], Einar Dale[7], Cecilia Marie Futsaether[1]*

[1]Norwegian University of Life Sciences, Faculty of Science and Technology, Ås, Norway

[2]Norwegian University of Science and Technology, Department of Circulation and Medical Imaging, Trondheim, Norway

[3]Oslo University Hospital, Department of Medical Physics, Oslo, Norway

[4]Maastricht University Medical Center, Department of Radiation Oncology (MAASTRO), Maastricht, The Netherlands

[5]GROW School for Oncology and Reproduction, Maastricht University Medical Center+, Maastricht, The Netherlands

[6]University of Oslo, Department of Physics, Oslo, Norway

[7]Oslo University Hospital, Department of Oncology, Oslo, Norway

*** Correspondence:**
Cecilia Marie Futsaether
cecilia.futsaether@nmbu.no

Appendix A. Patient and image data

Supplementary Table A1. Treatment characteristics.

Treatment	OUS (n = 139)	MAASTRO (n = 99)
	No. of patients (%)	
Radiotherapy[a]	139 (100)	99 (100)
Nimorazole[b]	132 (95)	0 (0)
Chemotherapy	100 (72)	26 (26)

OUS: Oslo University Hospital; MAASTRO: Maastro Clinic, Maastricht.
[a] 68–70 Gy to the high-risk clinical target volume in 1.8–2 Gy fractions.
[b] Hypoxic radiosensitizer.

Supplementary Table A2. PET/CT acquisition and reconstruction parameters.

Parameter	OUS (*n* = 139)	MAASTRO (*n* = 99)
Scanner	Siemens Biograph 16, Siemens Healthineers GmbH, Erlangen, Germany	Siemens Biograph 40, Siemens Healthineers GmbH, Erlangen, Germany
CT		
Scan mode	Helical (rotation time 0.5 s, pitch 0.75)	Helical (rotation time 1.0 s, pitch 0.8)
Peak tube voltage	120 kV	120 kV
Reconstructed slice thickness	2.00 mm	3.00 mm
Reconstruction kernel	B30f/B30s	B31s
Matrix size	512 × 512	512 × 512
Pixel size	0.98×0.98 mm^2	0.98×0.98 mm^2
	1.37×1.37 mm^2 (*n* = 26)	
	0.89×0.89 mm^2 (*n* = 1)	
	0.82×0.82 mm^2 (*n* = 1)	
Contrast agent	Visipaque 320 mg iodine/mL	Ultravist 300 mg iodine/mL
PET		
Reconstruction algorithm	OSEM, 4 iterations, 8 subsets	OSEM, 4 iterations, 8 subsets
Bed position overlap	25 %	25 %
Post reconstruction filter	Gaussian, FWHM 3.5 mm (*n* = 135)	Gaussian, FWHM 5.00 mm
	Gaussian, FWHM 5.0 mm (*n* = 2)	
	Gaussian, FWHM 2.0 mm (*n* = 2)	
Matrix size	256 × 256	256 × 256
Voxel size (*x*–*y*–*z*)	$2.66 \times 2.66 \times 2.00$ mm^3 (*n* = 91)	$2.67 \times 2.67 \times 3.00$ mm^3
	$1.77 \times 1.77 \times 2.00$ mm^3 (*n* = 20)	
	$2.66 \times 2.66 \times 5.00$ mm^3 (*n* = 20)	
	$2.66 \times 2.66 \times 1.00$ mm^3 (*n* = 3)	
	1.33 × 1.33 × 2.00 mm3 (*n* = 3)	
	$4.06 \times 4.06 \times 2.00$ mm^3 (*n* = 1)	
	$4.06 \times 4.06 \times 1.00$ mm^3 (*n* = 1)	

OUS: Oslo University Hospital; MAASTRO: Maastro Clinic, Maastricht; CT: computed tomography; PET: positron emission tomography; OSEM: Ordered Subset Expectations Maximization; FWHM: full width at half maximum.

Appendix B. Radiomics features extraction

All radiomics features used in this study were extracted using our in-house library *imskaper*[1]. The *imskaper* library can extract IBSI radiomics features using PyRadiomics (1) and ten additional 3D LBP features (2) for each modality/structure.

Due to the variation of head and neck nodal structures, we only extracted radiomics features from the primary tumors. All resampled PET and CT images were discretized before putting into the *imskaper* pipeline. Two binning methods (3) were used for discretization: based on the number of bins (bin counts) and based on the magnitude of bins (bin width). The selected binning options for CT images were (i) 16 bin counts and (ii) a bin width of 20, whereas these options for PET images were (iii) 4 bin counts and (iv) a bin width of 2. For each binning option, 14 shape features, 18 first-order features, 75 texture features and 10 LBP features were extracted, giving a total of 117 radiomics features. Since there were four binning options (two for each image modality), the original radiomics features contained 468 features.

Since all shape features depended solely on the primary tumor masks, the discretization process did not affect these values. Therefore, there were 42 duplicated shape features, which were then removed during data pre-processing (see Section 2.4). Similarly, LBP features depended on the input image modality and the primary tumor mask, resulting in the removal of 20 duplicated LBP features. In addition, 32 first-order features were also removed due to duplication. Thus, the radiomics features used in this study consisted of 374 features.

---

[1] Available at https://github.com/NMBU-Data-Science/imskaper

Appendix C. Models architectures and hyperparameters

In this study, we used the Python library scikit-learn[2] version 1.0.2 to train, validate and test the logistic regression model M1 and random forest M2. Therefore, if not mentioned, the hyperparameters of these models took the default values according to scikit-learn. For deep learning models M3-M7, we focused on optimizing hyperparameters relating to model complexity and loss function. Supplementary Table C1 shows the list of hyperparameters that were optimized during model training and validation. The selected values for these hyperparameters are shown in Supplementary Table C2.

All deep learning experiments were run using *deoxys*[3] version 0.1.11, our in-house Python framework for running deep-learning pipelines with emphasis on tasks relating to medical data. The code for running the full deep learning pipeline is available at https://github.com/huynhngoc/hnc-outcome-analysis.

The neural networks M3 (without interaction) and M4 (with interaction) are outlined in Supplementary Table C3 and C4. The interaction between nodes in model M4 was based on adding (Add layers) and multiplying (Multiply layers) nodes between different layers. Before the prediction layer, dropout with a rate of 25% was applied. Sigmoid activation function was also applied to the prediction layer, making the model outputs between 0 and 1.

While the input for the M5 model (PET/CT only) can be fed directly to the EfficientNet (4), models M6 (PET/CT and GTVp) and M7 (PET/CT, GTVp, and GTVn) needed some preliminary layers to emphasize the effect of the additional primary tumor GTVp and node GTVn masks, as shown in Supplementary Table C5. The outputs of these layers (namely input_1) were then fed into the scaled 3D EfficientNet.

The 3D EfficientNet (B1 complexity, as outline in GitHub[4]) in this study was down-scaled, making all convolutional layers have only half of the filters compared to the original model. The implementation of 3D EfficientNet was by replacing any 2D layers with 3D layers from the TensorFlow version of 2D EfficientNet.

To avoid the model being optimistic toward one metric, we used a weighted score for hyperparameters optimization. The score was mainly based on the validation AUC, MCC and positive class (class 1) F1 score. In addition, F1 score on the negative class (class 0) was also considered with a slightly lower weight. Since there were only few samples in each validation fold (27-28 samples), the risk that a model performed well on the validation but poorly on the training dataset or vice versa was high. Therefore, we included the training F1 score (class 1) to select models

---

[2] https://scikit-learn.org/1.0/modules/classes.html

[3] https://pypi.org/project/deoxys/

[4] https://gist.github.com/huynhngoc/fc385142dd9255343ae27c398a0ab843

that performed well on both training and validation data. The weighted score (score$_{weighted}$) is given by the following equation:

$$\text{score}_{\text{weighted}} = AUC_{val} + MCC_{val} + F1_{1_{val}} + 0.75 \cdot F1_{0_{val}} + 0.5 \cdot \sqrt{F1_{1_{train}}}$$

Supplementary Table C1. List of hyperparameters choices for training and validating machine learning models (M1 and M2), the fully connected neural networks (M3 and M4) and the convolutional neural networks (M5-M7).

ID	Model name	Hyperparameters	Values
M1	Logistic model	Solver	liblinear
		Regularizer	L1, L2
		C	0.01, 0.03, 0.09, 0.23, 0.62, 1.62, 4.48,…, 545 [a]
M2	Random forest	Number of Estimators	10, 20, 30, 40, …, 100
		Max Features	1, 2, 3, …, <number of input features>
M3	Neural Network (without interation)	Optimizer	Adam, SGD
		Learning rate	0.0001, 0.001, 0.01, 0.1
		Loss function	Binary Cross Entropy, F1 Loss
		First layer's number of nodes	16, 32, 64, 128
		Drop-out rate	0.1, 0.25, 0.3, 0.5
M4	Neural Network (with interaction)	Optimizer	Adam, SGD
		Learning rate	0.0001, 0.001, 0.01, 0.1
		Loss function	Binary Cross Entropy, F1 Loss
		First layers' number of nodes	4, 8, 16
		Drop-out rate	0.1, 0.25, 0.3, 0.5
M5-M7	EfficientNet (PET/CT only, PET/CT with GTVp, PET/CT with both GTVp and GTVn)	Base model	B0, B1, B2
		Scaled factor	0.25, 0.5, 1

[a] This is the geometric sequence starting from 0.01, with the common ratio of $10^{0.4}$.

Supplementary Table C2. List of selected hyperparameters for the machine learning models (M1 and M2), the fully connected neural networks (M3 and M4) and the convolutional neural networks (M5-M7).

Model	Input Group	RENT frequency	Hyperparameter name	Value for DFS	Value for OS
Logistic Model (M1)	Clinical factors (D1)	NA	Penalty	L2	L2
			C	0.01	0.01
		1 %	Penalty	L2	L2
			C	0.01	0.03
		50 %	Penalty	L2	L2
			C	0.23	0.01
	Radiomics data (D2)	NA	Penalty	L1	L1
			C	0.62	0.23
		1 %	Penalty	L2	L2
			C	0.62	0.03
		50 %	Penalty	L2	L1
			C	0.23	78.48
	Tabular data (D1+D2)	NA	Penalty	L1	L1
			C	0.23	0.23
		1 %	Penalty	L2	L2
			C	0.23	0.03
		50 %	Penalty	L2	L2
			C	0.62	0.01
Random forest (M2)	Clinical factors (D1)	NA	number of estimators	70	70
			max features	1	3
		1 %	number of estimators	20	30
			max features	1	4
		50 %	number of estimators	50	20
			max features	1	2
	Radiomics data (D2)	NA	number of estimators	90	80
			max features	7	11
		1 %	number of estimators	90	100
			max features	3	13
		50 %	number of estimators	80	80
			max features	1	4

	Tabular data (D1+D2)	NA	number of estimators	30	20
			max features	14	11
		1 %	number of estimators	80	60
			max features	11	1
		50 %	number of estimators	100	60
			max features	1	2
Neural Network (without interaction) (M3)			Optimizer	Adam	
			Learning rate	0.001	
			Loss function	Binary Cross Entropy	
			First layer's number of nodes	64	
			Drop-out rate	0.25	
Neural Network (with interaction) (M4)			Optimizer	Adam	
			Learning rate	0.001	
			Loss function	Binary Cross Entropy	
			First layers number of nodes	8	
			Drop-out rate	0.25	
EfficientNet (M5-M7)			Base model	B1	
			Scaled factor	0.5	

Supplementary Table C3. The fully connected neural network (M3) architecture.

Name	Inputs	Output Shape
Dense1	Input	64
Dense2	Dense1	32
Dense3	Dense2	16
Dense4	Dense3	8
Dense5	Dense4	8
Prediction	Dense5	1

Supplementary Table C4. The fully connected neural network (M4) with interaction architecture.

Name	Inputs	Output Shape
Path1_Dense1	Input	8
Path2_Dense1	Input	8
Path3_Dense1	Input	8
Path1_Dense2	Path1_Dense1	8
Path2_Dense2	Path2_Dense1	8
Path3_Dense2	Path3_Dense1	8
Path1_Add	Path1_Dense1, Path1_Dense2	8
Path2_Add	Path2_Dense1, Path2_Dense2	8
Path3_Add	Path3_Dense1, Path3_Dense2	8
Multiply_12	Path1_Add, Path2_Add,	8
Multiply_23	Path2_Add, Path3_Add,	8
Multiply_13	Path1_Add, Path3_Add,	8
Multiply_123	Path1_Add, Path2_Add, Path3_Add,	8
Concatenate	Input, Path1_Add, Path2_Add, Path3_Add, Multiply_12, Multiply_23, Multiply_13, Multiply_123	56 + <input shape>
Dense1	Concatenate	32
Dense2	Dense1	16
Prediction	Dense2	1

Supplementary Table C5. The architecture of the first few layers in the 3D EfficientNet M6 and M7.

**Name**	**Input**	**Output Shape**
	EfficientNet M6 (CT, PET, GTVp)	
CT_tumor_area	CT, GTVp	173×191×265×1
PET_tumor_area	PET, GTVp	173×191×265×1
input_1	Input (PET, CT, GTVp), CT_tumor_area, PET_tumor_area	173×191×265×5
	EfficientNet M7 (CT, PET, GTVp, GTVn)	
CT_tumor_area	CT, GTVp	173×191×265×1
PET_tumor_area	PET, GTVp	173×191×265×1
CT_node_area	CT, GTVp	173×191×265×1
PET_node_area	PET, GTVp	173×191×265×1
input_1	Input (PET, CT, GTVp, GTVn), CT_tumor_area, PET_tumor_area, CT_node_area, PET_node_area	173×191×265×8

Appendix D. Performance metric definitions

As outlined in Section 2.8, the following five main performance metrics were computed: (i) Accuracy, (ii) the area under the receiver operating characteristic curve (AUC), (iii) Matthew's correlation coefficient (MCC), and F1 score on class 1 (iv) and class 0 (v) separately. In addition, the precision, recall, and specificity were computed.

Seven of the above eight metrics are based directly on the counts found in a binary confusion matrix, i.e., true positives (*TP*), false negatives (*FN*), true negatives (*TN*), and false positives (*FP*). The basic metric of success is accuracy:

$$\text{accuracy} = \frac{TP+TN}{TP+FN+TN+FP} \ ,$$

i.e., the proportion correctly classified patients. Further, the precision (proportion correctly classified among positive predictions), recall (proportion correctly classified positive samples), and specificity (proportion correctly classified negative samples) are defined as:

$$\text{precision} = \frac{TP}{TP+FP} \ , \qquad \text{recall} = \frac{TP}{TP+FN} \ , \qquad \text{specificity}= \frac{TN}{TN+FP}.$$

Furthermore, we report the F1 score for the positive class $F1_1$ (event, class 1) and negative class $F1_0$ (no event, class 0), given by:

$$F1_1 = \frac{2TP}{2TP+FN+FP} \ , \qquad F1_0 = \frac{2TN}{2TN+FN+FP} \ .$$

The latter is included since the F1 score is an asymmetric metric disregarding *TN* for class 1 and *TP* for class 0. F1 is the harmonic mean of precision and recall.

We also include the MCC, which is defined as (5):

$$\text{MCC} = \frac{TP \times TN - FP \times FN}{\sqrt{(TP+FP)(TP+FN)(TN+FP)(TN+FN)}} \ .$$

MCC is a symmetric correlation measure shown to be more robust than F1 and ACC (6). This means that switching positive (event) and negative (no event) class labels gives the same MCC. The MCC scales from -1 to 1, where 1 is perfect correlation, 0 is random prediction and -1 is reversing the classes in prediction. Since all other metrics used in this study scale from 0 to 1, we rescaled MCC to the interval 0 to 1 for easier comparison, as follows:

$$\text{MCC}_{\text{scaled}} = \frac{MCC+1}{2} \ .$$

Finally, we included the AUC, which is another measure of success ranging from 0 to 1 where 0.5 is random prediction. AUC is based on the receiver operating characteristic curve (ROC), which is produced by tracing the true positive rate (TPR, which is also referred to as recall) as a function of the false positive rate (FPR):

$$\text{TPR} = \frac{TP}{TP+FN} \ , \qquad \text{FPR} = \frac{FP}{FP+TN} \ .$$

from right to left (1 to 0) where each occurrence of a negative sample (class 0) results in a drop in the curve, ideally clustered at the very left of the plot. However, according to a recent simulation study, the MCC should replace the ROC as the standard metric for assessing binary classification as ROC,

in similarity with the F1-score, can in some cases be overly optimistic (7). For simplicity, the scaled MCC (MCC_{scaled}) is referred to as MCC throughout the main text of our study.

Appendix E. RENT selected features

Supplementary Table E1. Features selected by RENT for predicting DFS.

Feature name	Frequency (%)
**Clinical factors & PET parameters (D1)**	
hpv_related	98
uicc8_III-IV	89
pack_years	41
cavum_oris	39
oropharynx	37
larynx	4
charlson	2
female	1
age	1
SUVpeak	1
**Radiomics data (D2)**	
shape_Sphericity	95
LBP_102_PET	95
shape_Elongation	69
glszm_SmallAreaLowGrayLevelEmphasis_CT_c16	63
LBP_201_PET	48
glszm_GrayLevelNonUniformityNormalized_PET_c04	31
LBP_201_CT	12
shape_Flatness	9
glrlm_ShortRunLowGrayLevelEmphasis_PET_c04	9
glszm_ZoneEntropy_PET_b2	8
LBP_021_PET	4
glszm_SizeZoneNonUniformityNormalized_PET_b2	3
glszm_ZoneEntropy_PET_c04	2
gldm_LargeDependenceLowGrayLevelEmphasis_d_1_CT_c16	2
glszm_SmallAreaHighGrayLevelEmphasis_PET_b2	2
glcm_MaximumProbability_d_1_PET_b2	1
LBP_003_CT	1
LBP_300_CT	1
glszm_GrayLevelVariance_PET_c04	1

LBP_102_CT	1
glrlm_ShortRunHighGrayLevelEmphasis_PET_c04	1
glcm_SumSquares_d_1_PET_c04	1
glrlm_HighGrayLevelRunEmphasis_PET_c04	1
first_order_Minimum_PET	1
glszm_GrayLevelNonUniformityNormalized_PET_b2	1
**All tabular data (D1+D2)**	
shape_Sphericity	98
shape_Elongation	95
LBP_102_PET	94
glszm_SmallAreaLowGrayLevelEmphasis_CT_c16	85
LBP_201_PET	68
hpv_related	55
glszm_GrayLevelNonUniformityNormalized_PET_c04	49
uicc8_III-IV	47
shape_Flatness	18
glszm_ZoneEntropy_PET_b2	17
glrlm_ShortRunLowGrayLevelEmphasis_PET_c04	16
glszm_SmallAreaHighGrayLevelEmphasis_PET_b2	13
cavum_oris	12
LBP_201_CT	11
age	10
female	9
LBP_021_PET	8
larynx	5
hypopharynx	5
glszm_SizeZoneNonUniformityNormalized_CT_b20	3
glrlm_HighGrayLevelRunEmphasis_PET_c04	3
glszm_ZoneEntropy_PET_c04	2
glcm_SumSquares_d_1_PET_c04	2
glcm_ClusterProminence_d_1_PET_b2	2
glszm_SizeZoneNonUniformityNormalized_PET_b2	2
glszm_SizeZoneNonUniformity_PET_b2	2
glszm_SmallAreaLowGrayLevelEmphasis_PET_b2	2

glszm_GrayLevelNonUniformityNormalized_PET_b2	2
histgrade_high	2
glcm_Imc1_d_1_CT_b20	1
glcm_MaximumProbability_d_1_PET_b2	1
first_order_Kurtosis_PET	1
gldm_LargeDependenceLowGrayLevelEmphasis_d_1_CT_c16	1
LBP_102_CT	1
glrlm_ShortRunHighGrayLevelEmphasis_PET_c04	1
glcm_JointEnergy_d_1_CT_b20	1
gldm_DependenceEntropy_d_1_PET_c04	1
first_order_Skewness_CT	1
LBP_021_CT	1
glcm_MCC_d_1_CT_c16	1
glcm_ClusterTendency_d_1_PET_c04	1
glcm_MCC_d_1_CT_b20	1

Supplementary Table E2. Features selected by RENT for predicting OS.

Feature name	Frequency (%)
**Clinical factors & PET parameters (D1)**	
uicc8_III-IV	100
hpv_related	95
pack_years	47
oropharynx	36
age	6
cavum_oris	4
charlson	4
**Radiomics data (D2)**	
shape_Sphericity	100
glcm_JointAverage_d_1_CT_c16	79
glcm_SumAverage_d_1_CT_c16	79
shape_MajorAxisLength	57
first_order_Maximum_CT	35
glrlm_HighGrayLevelRunEmphasis_PET_c04	34
shape_Maximum3DDiameter	31
glcm_ClusterShade_d_1_PET_b2	25
gldm_LargeDependenceLowGrayLevelEmphasis_d_1_CT_c16	17
gldm_LargeDependenceHighGrayLevelEmphasis_d_1_CT_c16	12
glrlm_LowGrayLevelRunEmphasis_PET_c04	7
glcm_JointAverage_d_1_PET_c04	7
glcm_SumAverage_d_1_PET_c04	7
LBP_102_PET	7
first_order_Minimum_PET	6
glcm_Autocorrelation_d_1_PET_c04	5
glszm_ZoneEntropy_CT_b20	5
glrlm_ShortRunHighGrayLevelEmphasis_PET_c04	4
shape_Maximum2DDiameterSlice	3
gldm_DependenceVariance_d_1_CT_b20	3
first_order_Skewness_PET	3
LBP_210_CT	2
first_order_Skewness_CT	2

glszm_GrayLevelNonUniformityNormalized_CT_b20	2
ngtdm_Busyness_d_1_PET_c04	2
gldm_HighGrayLevelEmphasis_d_1_CT_c16	2
glcm_Autocorrelation_d_1_CT_c16	2
first_order_Range_CT	1
ngtdm_Busyness_d_1_CT_b20	1
ngtdm_Busyness_d_1_PET_b2	1
shape_Elongation	1
gldm_HighGrayLevelEmphasis_d_1_PET_c04	1
**All tabular data (D1+D2)**	
shape_Sphericity	100
uicc8_III-IV	88
hpv_related	86
oropharynx	12
pack_years	6
cavum_oris	2
Age	2

Appendix F. Model performances

A. CLINICAL (D1)

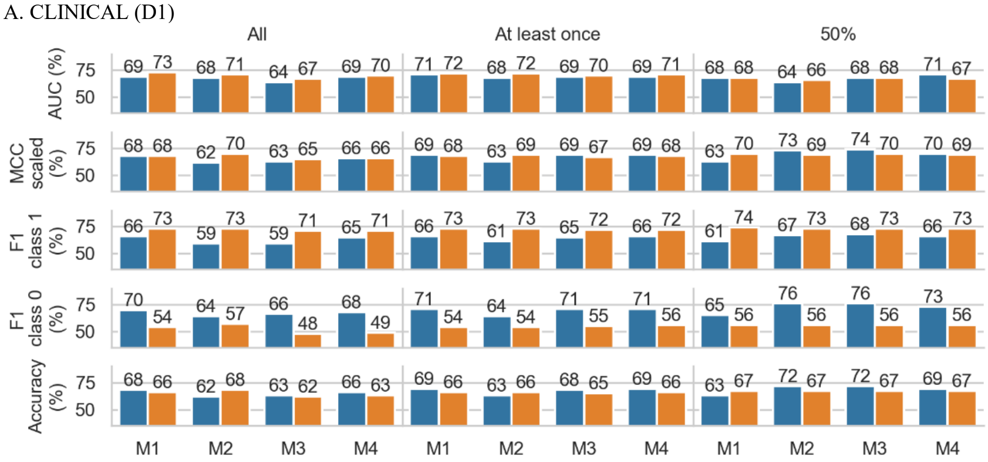

B. RADIOMICS (D2)

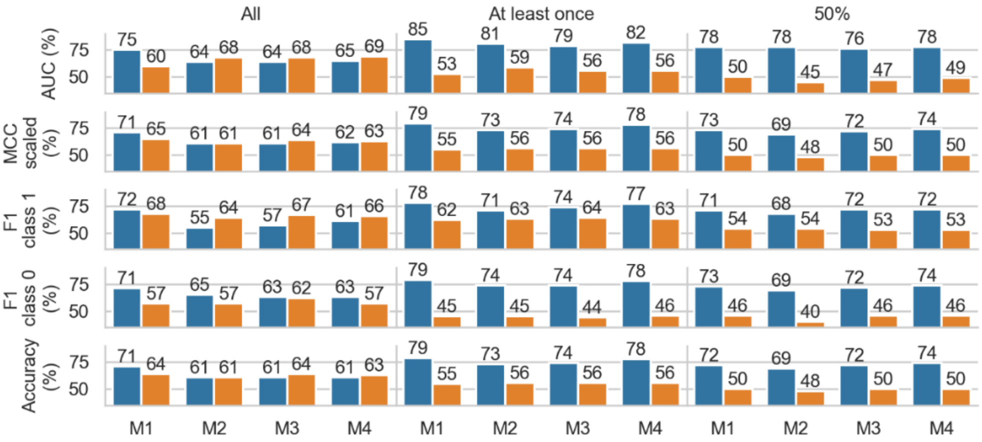

C. CLINICAL + RADIOMICS (D1+D2)

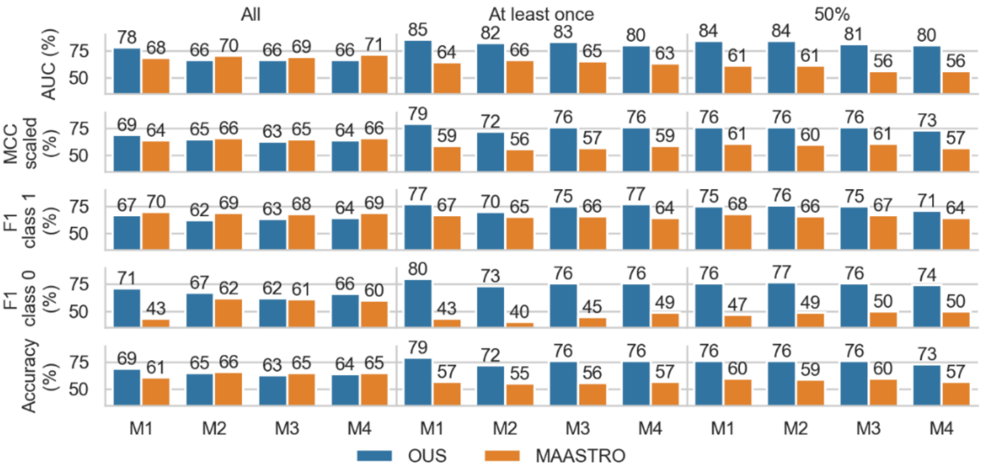

Supplementary Figure F1. Median performance metrics for prediction of DFS by tabular based models (M1-M4) trained on (A) clinical data (D1), (B) radiomics features (D2) and (C) all tabular data (D1+D2). All metrics were calculated from bootstrap sampling the OUS and MAASTRO datasets to maintain the 1:1 ratio between class 1 (event occurrence) and class 0. The column group indicates which features were selected as input: all features were used in the first column group, while models in the second and third column group trained on features that were selected by RENT once or with 50% frequency, respectively.

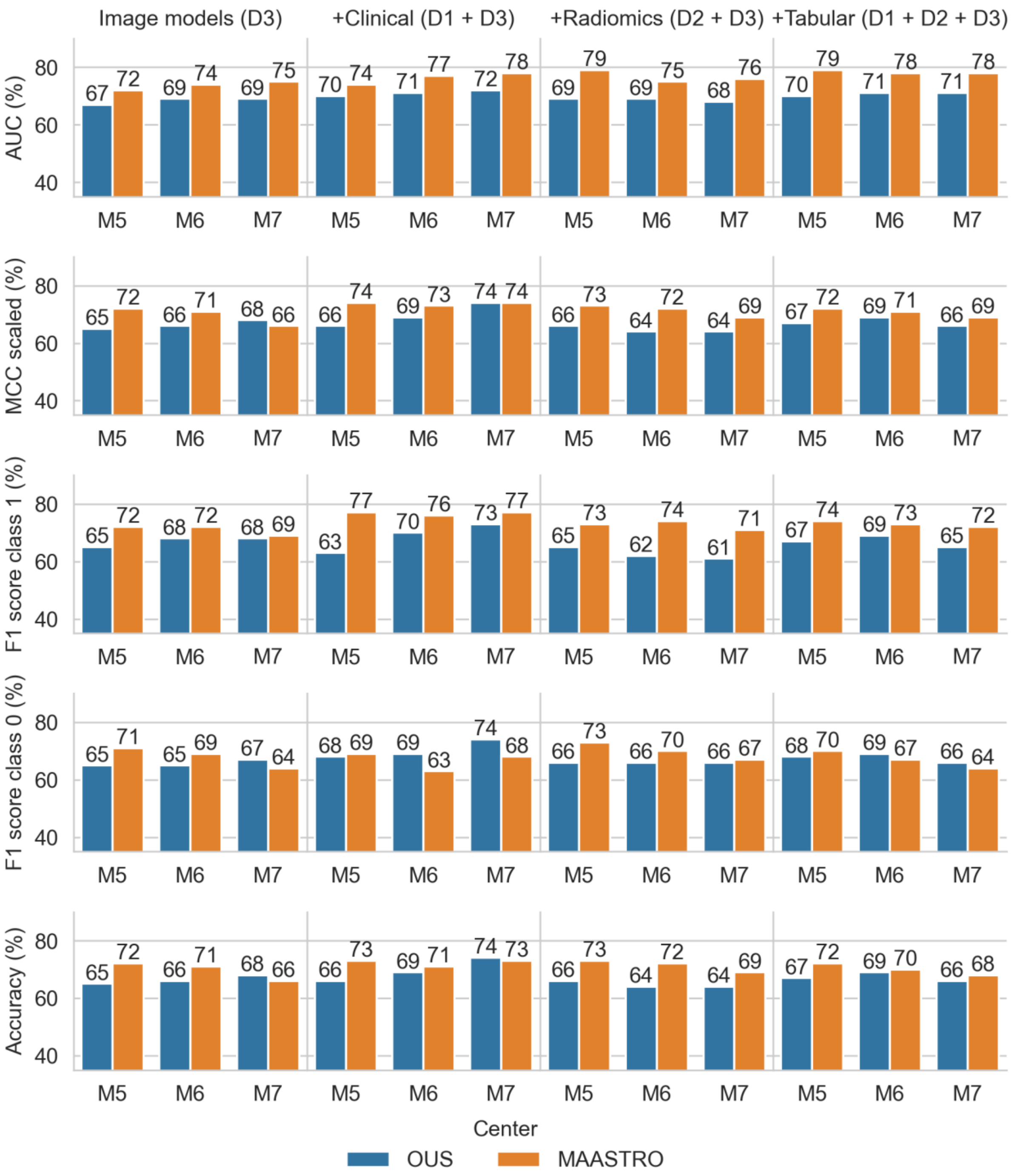

Supplementary Figure F2. Median performance metrics for prediction of DFS by CNN models (M5-M7). All metrics were calculated from bootstrap sampling the OUS and MAASTRO datasets to maintain the 1:1 ratio between class 1 (event occurrence) and class 0. The column groups show (first column) models trained on D3 only (images + GTV masks), and (second to fourth columns) models trained on D3 combined (via ensemble averaging) with models trained on clinical data (D1), radiomics data (D2) and all tabular data (D1+D2).

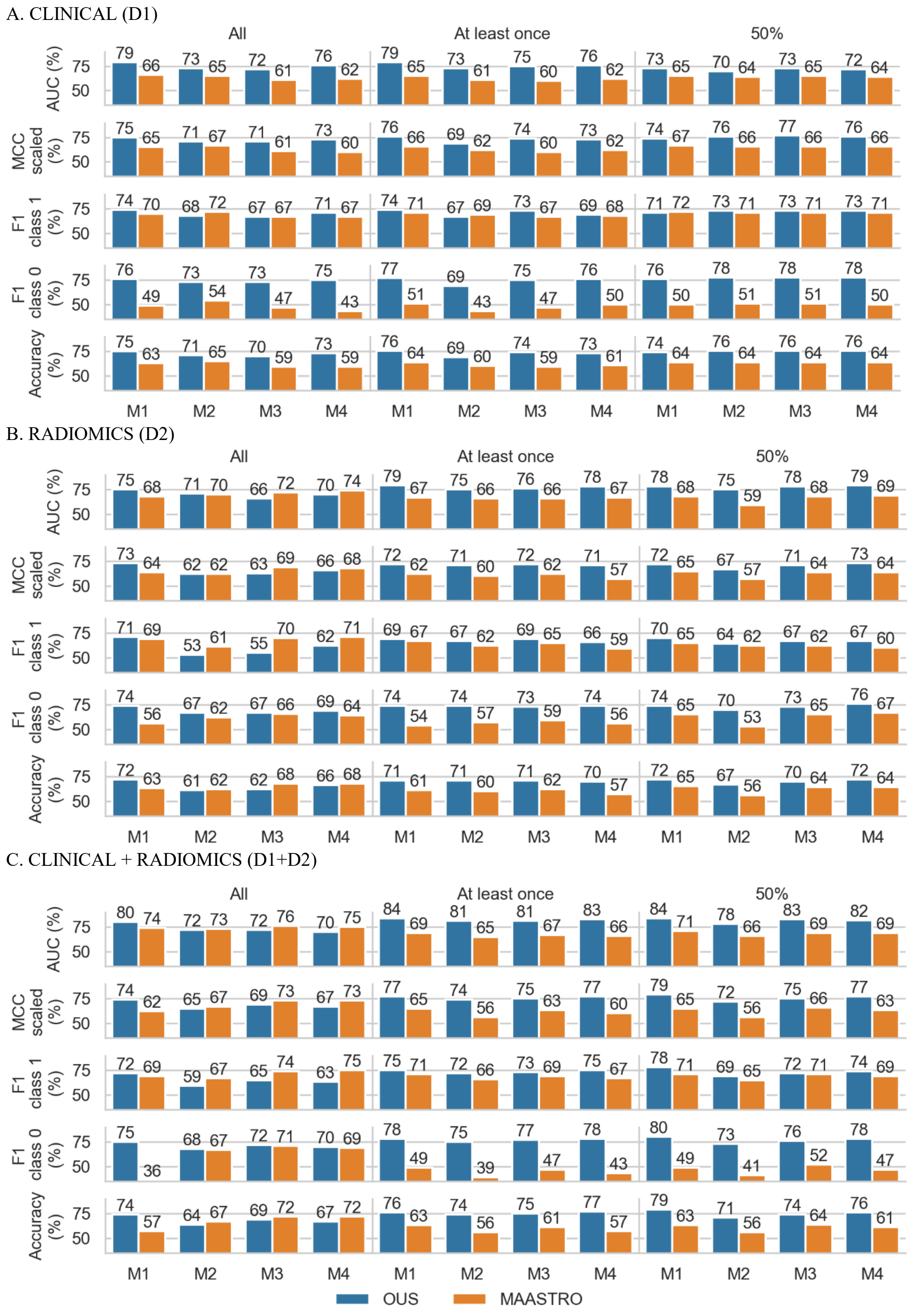

Supplementary Figure F3. Median performance metrics for prediction of OS by tabular based models (M1-M4) trained on (A) Clinical data (D1), (B) Radiomics features (D2) and (C) all tabular data (D1+D2). All metrics were calculated from bootstrap sampling the OUS and MAASTRO datasets to maintain the 1:1 ratio between class 1 (event occurrence) and class 0. The column group indicates which features were selected as input: all features were used in the first column group, while models in the second and third column group trained on features that were selected by RENT once or with 50% frequency, respectively.

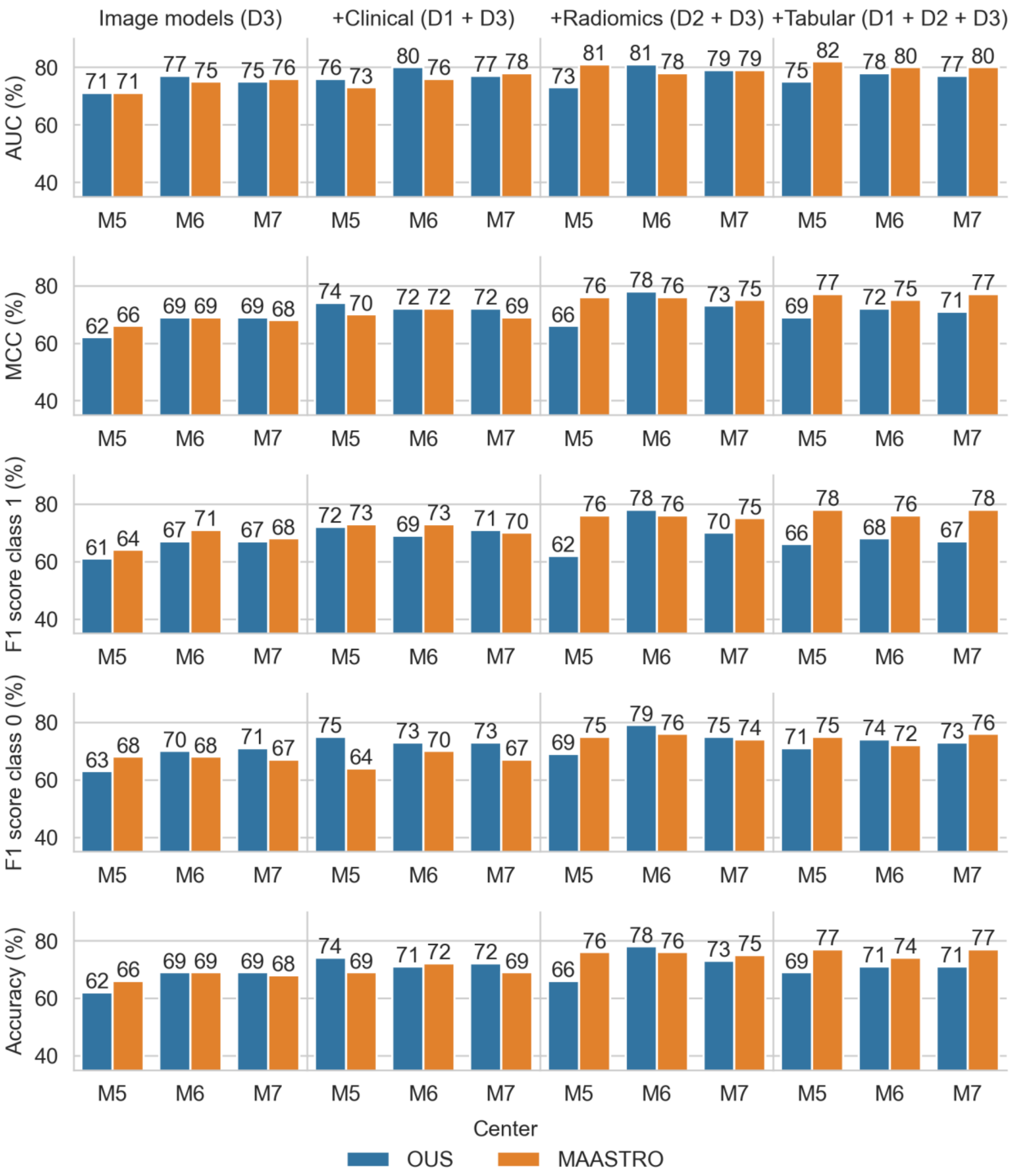

Supplementary Figure F4. Median performance metrics for prediction of OS by CNN models (M5-M7). All metrics were calculated from bootstrap sampling the OUS and MAASTRO datasets to maintain the 1:1 ratio between class 1 (event occurrence) and class 0. The column groups show (first column) models trained on D3 only (images + GTV masks), and (second to fourth columns) models trained on D3 combined (via ensemble averaging) with models trained on clinical data (D1), radiomics data (D2) and all tabular data (D1+D2).

Supplementary Table F1. Median performance metrics for prediction of DFS by all models (M1-M7) from clinical data D1, radiomics data D2, image data D3 or combination of these input data. All metrics were calculated from bootstrap sampling the OUS and MAASTRO datasets to maintain the 1:1 ratio between class 1 (event occurrence) and class 0.

Data	RENT selection	Model	AUC		MCC		F1 score class 1		F1 score class 0		Precision		Recall		Specificity		Accuracy	
			OUS	MAASTRO	OUS	MAASTRO	OUS	MAASTRO	OUS	MAASTRO	OUS	MAASTRO	OUS	MAASTRO	OUS	MAASTRO	OUS	MAASTRO
D1	All	M1	69	73	68	68	66	73	70	54	72	61	60	91	76	40	68	66
		M2	68	71	62	70	59	73	64	57	64	62	55	89	68	44	62	68
		M3	64	67	63	65	59	71	66	48	66	57	54	89	72	34	63	62
		M4	69	70	66	66	65	71	68	49	68	59	62	91	71	34	66	63
	At least once	M1	71	72	69	68	66	73	71	54	74	61	60	91	78	40	69	66
		M2	68	72	63	69	61	73	64	54	64	61	57	91	66	40	63	66
		M3	69	70	69	67	65	72	71	55	73	61	57	87	78	42	68	65
		M4	69	71	69	68	66	72	71	56	74	62	60	87	78	44	69	66
	50 %	M1	68	68	63	70	61	74	65	56	65	62	57	91	68	42	63	67
		M2	64	66	73	69	67	73	76	56	83	62	56	91	88	42	72	67
		M3	68	68	74	70	68	73	76	56	83	62	56	91	88	42	72	67
		M4	71	67	70	69	66	73	73	56	77	62	56	91	82	42	69	67
D2	All	M1	75	60	71	65	72	68	71	57	72	61	71	77	72	51	71	64
		M2	64	68	61	61	55	64	65	57	64	61	48	67	73	55	61	61
		M3	64	68	61	64	57	67	63	62	62	63	54	71	66	59	61	64
		M4	65	69	62	63	61	66	63	57	63	61	57	73	65	53	61	63
	At least once	M1	85	53	79	55	78	62	79	45	81	54	75	71	82	36	79	55
		M2	81	59	73	56	71	63	74	45	77	55	65	75	79	36	73	56
		M3	79	56	74	56	74	64	74	44	75	55	74	77	75	34	74	56
		M4	82	56	78	56	77	63	78	46	80	55	75	75	81	38	78	56

	50 %	M1	78	50	73	50	71	54	73	46	75	50	68	56	76	42	72	50
		M2	78	45	69	48	68	54	69	40	69	49	68	59	69	34	69	48
		M3	76	47	72	50	72	53	72	46	73	50	70	56	73	42	72	50
		M4	78	49	74	50	72	53	74	46	76	50	68	56	78	42	74	50
D1+D2	All	M1	78	68	69	64	67	70	71	43	73	56	61	91	78	30	69	61
		M2	66	70	65	66	62	69	67	62	68	63	56	75	72	55	65	66
		M3	66	69	63	65	63	68	62	61	63	63	62	73	63	57	63	65
		M4	66	71	64	66	64	69	66	60	66	63	61	77	68	53	64	65
	At least once	M1	85	64	79	59	77	67	80	43	83	55	71	83	85	32	79	57
		M2	82	66	72	56	70	65	73	40	75	54	65	81	78	30	72	55
		M3	83	65	76	57	75	66	76	45	78	55	71	79	79	34	76	56
		M4	80	63	76	59	77	64	76	49	76	56	77	75	75	40	76	57
	50 %	M1	84	61	76	61	75	68	76	47	77	56	72	85	78	36	76	60
		M2	84	61	76	60	76	66	77	49	78	56	74	79	79	40	76	59
		M3	81	56	76	61	75	67	76	50	78	57	72	79	79	40	76	60
		M4	80	56	73	57	71	64	74	50	76	56	67	73	78	42	73	57
D3		M5	67	72	65	72	65	72	65	71	65	72	65	71	66	71	65	72
		M6	69	74	66	71	68	72	65	69	65	69	70	75	62	67	66	71
		M7	69	75	68	66	68	69	67	64	68	65	68	71	66	61	68	66
D1 + D3		M5	70	74	66	74	63	77	68	69	69	69	57	85	72	61	66	73
		M6	71	77	69	73	70	76	69	63	69	65	71	91	68	51	69	71
		M7	72	78	74	74	73	77	74	68	75	68	71	87	76	59	74	73
D2 + D3		M5	69	79	66	73	65	73	66	73	67	73	64	73	68	73	66	73
		M6	69	75	64	72	62	74	66	70	66	70	57	79	69	67	64	72
		M7	68	76	64	69	61	71	66	67	66	67	56	75	71	63	64	69
D1 + D2 + D3		M5	70	79	67	72	67	74	68	70	68	70	65	77	69	67	67	72
		M6	71	78	69	71	69	73	69	67	70	67	69	79	69	61	69	70
		M7	71	78	66	69	65	72	66	64	67	66	64	79	68	59	66	68

OUS: Oslo University Hospital; MAASTRO: Maastro Clinic, Maastricht; RENT: the feature selection library using repeated elastic net technique; AUC: area under the receiver operating characteristic curve; MCC: Matthew's correlation coefficient

Supplementary Table F2. Median performance metrics for prediction of OS by all models (M1-M7) from clinical data D1, radiomics data D2, image data D3 or combination of these input data. All metrics were calculated from bootstrap sampling the OUS and MAASTRO datasets to maintain the 1:1 ratio between class 1 (event occurrence) and class 0.

Data	RENT selection	Model	AUC		MCC		F1 score class 1		F1 score class 0		Precision		Recall		Specificity		Accuracy	
			OUS	MAASTRO	OUS	MAASTRO	OUS	MAASTRO	OUS	MAASTRO	OUS	MAASTRO	OUS	MAASTRO	OUS	MAASTRO	OUS	MAASTRO
D1	All	M1	79	66	75	65	74	70	76	49	78	59	70	89	81	36	75	63
		M2	73	65	71	67	68	72	73	54	75	61	62	87	79	42	71	65
		M3	72	61	71	61	67	67	73	47	75	56	61	81	79	36	70	59
		M4	76	62	73	60	71	67	75	43	78	56	64	85	82	32	73	59
	At least once	M1	79	65	76	66	74	71	77	51	80	59	69	89	82	36	76	64
		M2	73	61	69	62	67	69	69	43	70	56	64	87	72	30	69	60
		M3	75	60	74	60	73	67	75	47	78	56	68	81	81	36	74	59
		M4	76	62	73	62	69	68	76	50	80	57	61	83	85	38	73	61
	50 %	M1	73	65	74	67	71	72	76	50	80	59	65	89	83	36	74	64
		M2	70	64	76	66	73	71	78	51	84	59	64	89	88	38	76	64
		M3	73	65	77	66	73	71	78	51	84	59	65	89	88	36	76	64
		M4	72	64	76	66	73	71	78	50	84	59	64	89	88	36	76	64
D2	All	M1	75	68	73	64	71	69	74	56	76	60	67	81	79	46	72	63
		M2	71	70	62	62	53	61	67	62	67	63	44	59	79	63	61	62
		M3	66	72	63	69	55	70	67	66	68	67	47	75	78	63	62	68
		M4	70	74	66	68	62	71	69	64	71	65	54	79	78	57	66	68
	At least once	M1	79	67	72	62	69	67	74	54	76	59	64	77	81	46	71	61
		M2	75	66	71	60	67	62	74	57	77	60	60	65	82	55	71	60
		M3	76	66	72	62	69	65	73	59	75	61	64	69	79	55	71	62
		M4	78	67	71	57	66	59	74	56	79	57	55	59	85	57	70	57
	50 %	M1	78	68	72	65	70	65	74	65	76	65	65	65	79	65	72	65
		M2	75	59	67	57	64	62	70	53	71	56	57	67	76	48	67	56

		M3	78	68	71	64	67	62	73	65	76	66	60	56	82	71	70	64
		M4	79	69	73	64	67	60	76	67	83	67	55	55	89	73	72	64
D1+D2	All	M1	80	74	74	62	72	69	75	36	77	55	68	91	81	24	74	57
		M2	72	73	65	67	59	67	68	67	69	67	51	67	78	67	64	67
		M3	72	76	69	73	65	74	72	71	74	71	56	77	79	69	69	72
		M4	70	75	67	73	63	75	70	69	73	69	55	83	79	63	67	72
	At least once	M1	84	69	77	65	75	71	78	49	81	59	70	89	83	34	76	63
		M2	81	65	74	56	72	66	75	39	78	54	67	83	81	28	74	56
		M3	81	67	75	63	73	69	77	47	80	57	67	85	83	34	75	61
		M4	83	66	77	60	75	67	78	43	82	56	69	85	85	32	77	57
	50 %	M1	84	71	79	65	78	71	80	49	82	59	75	89	83	34	79	63
		M2	78	66	72	56	69	65	73	41	76	54	62	81	81	30	71	56
		M3	83	69	75	66	72	71	76	52	80	60	67	87	82	40	74	64
		M4	82	69	77	63	74	69	78	47	82	57	68	86	85	34	76	61
D3		M5	71	71	62	66	61	64	63	68	63	69	60	59	65	73	62	66
		M6	77	75	69	69	67	71	70	68	72	68	62	73	75	67	69	69
		M7	75	76	69	68	67	68	71	67	72	68	62	67	75	69	69	68
D1 + D3		M5	76	73	74	70	72	73	75	64	78	65	66	83	81	55	74	69
		M6	80	76	72	72	69	73	73	70	76	70	62	77	79	67	71	72
		M7	77	78	72	69	71	70	73	67	74	67	68	73	76	65	72	69
D2 + D3		M5	73	81	66	76	62	76	69	75	70	76	55	77	76	75	66	76
		M6	81	78	78	76	78	76	79	76	81	76	75	77	81	75	78	76
		M7	79	79	73	75	70	75	75	74	78	75	65	75	81	76	73	75
D1 + D2 + D3		M5	75	82	69	77	66	78	71	75	72	74	61	81	76	71	69	77
		M6	78	80	72	75	68	76	74	72	79	71	59	83	84	67	71	74
		M7	77	80	71	77	67	78	73	76	77	75	59	81	82	73	71	77

OUS: Oslo University Hospital; MAASTRO: Maastro Clinic, Maastricht; RENT: the feature selection library using repeated elastic net technique; AUC: area under the receiver operating characteristic curve; MCC: Matthew's correlation coefficient

References

1. van Griethuysen JJM, Fedorov A, Parmar C, Hosny A, Aucoin N, Narayan V, et al. Computational Radiomics System to Decode the Radiographic Phenotype. Cancer Research. 2017;77(21):e104-e7.

2. Montagne C, Kodewitz A, Vigneron V, Giraud V, Lelandais S, editors. 3D Local Binary Pattern for PET image classification by SVM, Application to early Alzheimer disease diagnosis. 6th International Conference on Bio-Inspired Systems and Signal Processing (BIOSIGNALS 2013); 2013.

3. Zwanenburg A, Vallières M, Abdalah MA, Aerts HJWL, Andrearczyk V, Apte A, et al. The Image Biomarker Standardization Initiative: Standardized Quantitative Radiomics for High-Throughput Image-based Phenotyping. Radiology. 2020;295(2):328-38.

4. Tan MX, Le QV. EfficientNet: Rethinking Model Scaling for Convolutional Neural Networks. Pr Mach Learn Res. 2019;97.

5. Matthews BW. Comparison of the predicted and observed secondary structure of T4 phage lysozyme. Biochim Biophys Acta. 1975;405(2):442-51.

6. Chicco D, Jurman G. The advantages of the Matthews correlation coefficient (MCC) over F1 score and accuracy in binary classification evaluation. BMC Genomics. 2020;21(1):6.

7. Chicco D, Jurman G. The Matthews correlation coefficient (MCC) should replace the ROC AUC as the standard metric for assessing binary classification. Biodata Min. 2023;16(1).
